# Identification of intermediate-sized deletions and inference of their impact on gene expression in a human population

**DOI:** 10.1186/s13073-019-0656-4

**Published:** 2019-07-24

**Authors:** Jing Hao Wong, Daichi Shigemizu, Yukiko Yoshii, Shintaro Akiyama, Azusa Tanaka, Hidewaki Nakagawa, Shu Narumiya, Akihiro Fujimoto

**Affiliations:** 1 Department of Drug Discovery Medicine, Kyoto University Graduate School of Medicine, Kyoto, Japan; 2 Department of Human Genetics, Graduate School of Medicine, The University of Tokyo, Tokyo, Japan; 3 Medical Genome Center, National Center for Geriatrics and Gerontology, Obu, Japan; 4 Laboratory for Medical Science Mathematics, RIKEN Center for Integrative Medical Science, Yokohama, Japan; 5 Department of Medical Science Mathematics, Medical Research Institute, Tokyo Medical and Dental University (TMDU), Tokyo, Japan; 6 Laboratory for Cancer Genomics, RIKEN Center for Integrative Medical Science, Yokohama, Japan

**Keywords:** Intermediate-sized deletion, Expression quantitative trait loci (eQTL), Genomic imputation, Long-read sequencing

## Abstract

**Background:**

Next-generation sequencing has allowed for the identification of different genetic variations, which are known to contribute to diseases. Of these, insertions and deletions are the second most abundant type of variations in the genome, but their biological importance or disease association is not well-studied, especially for deletions of intermediate sizes.

**Methods:**

We identified intermediate-sized deletions from whole-genome sequencing (WGS) data of Japanese samples (*n* = 174) with a novel deletion calling method which considered multiple samples. These deletions were used to construct a reference panel for use in imputation. Imputation was then conducted using the reference panel and data from 82 publically available Japanese samples with gene expression data. The accuracy of the deletion calling and imputation was examined with Nanopore long-read sequencing technology. We also conducted an expression quantitative trait loci (eQTL) association analysis using the deletions to infer their functional impacts on genes, before characterizing the deletions causal for gene expression level changes.

**Results:**

We obtained a set of polymorphic 4378 high-confidence deletions and constructed a reference panel. The deletions were successfully imputed into the Japanese samples with high accuracy (97.3%). The eQTL analysis identified 181 deletions (4.1%) suggested as causal for gene expression level changes. The causal deletion candidates were significantly enriched in promoters, super-enhancers, and transcription elongation chromatin states. Generation of deletions in a cell line with the CRISPR-Cas9 system confirmed that they were indeed causative variants for gene expression change. Furthermore, one of the deletions was observed to affect the gene expression levels of a gene it was not located in.

**Conclusions:**

This paper reports an accurate deletion calling method for genotype imputation at the whole genome level and shows the importance of intermediate-sized deletions in the human population.

**Electronic supplementary material:**

The online version of this article (10.1186/s13073-019-0656-4) contains supplementary material, which is available to authorized users.

## Background

Next-generation sequencing (NGS) technology enables us to detect various types of genetic variations such as single nucleotide variants (SNVs), insertions and deletions (indels), copy number variants, and structural variants (SVs) [1, 2]. A number of human genetic studies have revealed that many variants are associated with human diseases and contribute to disease risk prediction, as well as to understanding the molecular mechanism of diseases [3, 4]. Therefore, identification of genetic variations and inferring their functional impacts is one of the most important issues in human genetics studies.

Among genetic variations, SNVs are the most abundant and well-studied type of variants [3–5]. Besides SNVs, SVs have also been shown to be functionally important for disease associations. Small insertions and deletions (indels), which are also abundant within the human genome [5], and copy number variants (CNVs), which are typically > 1 kbp in length, were reported to be associated with a myriad of diseases including Mendelian diseases [6], as well as autism spectrum disease (ASD) [7], schizophrenia [7], and Alzheimer’s disease [8]. As SVs are typically large in size compared to SNVs, it stands to reason that their functional impact would be greater compared to SNVs or small indels. It is thus clear that more detailed analyses of such SVs could provide us with important information for future disease association studies.

Although the association of small indels and CNVs with disease and trait associations has been widely investigated, there have been limited studies of the functional impacts of other types of SVs. Previous studies have suggested that thousands of intermediate-sized deletions (30–5000 bp) exist per individual [9, 10], and we can expect that these deletions are likely to cause changes to the genome structure or contain variants that have greater functional impacts compared to SNVs. Indeed, it has also been reported that such intermediate-sized deletions can be associated with not only rare or low-frequency diseases [11, 12], but also more common neurological disorders such as Alzheimer’s disease [13] and common diseases such as prostate [14] and breast cancer [15]. This further demonstrates the importance of such intermediate-sized deletions in the genome.

However, studies of intermediate-sized deletions on a whole genome level are limited, and methods for the application of such intermediate-sized deletions have yet to be established. One reason would be the difficulty in accurately identifying such deletions. For variant calling, the accuracy of detection is extremely important [16]; however, unlike the detection of SNVs and CNVs by genotyping arrays, large-scale genotyping of intermediate-sized deletions with a genotyping array is not possible. Even when using whole-genome sequencing (WGS), repetitive sequences in the non-coding regions would hinder accurate detection of intermediate-sized deletions. Therefore, the development of appropriate methods and their evaluation are necessary for detecting intermediate-sized deletions at the whole genome level.

Recent large-scale whole-genome sequencing studies have identified many SVs and have also sought to analyze their association with gene expression [9, 17]. SVs and indels have been reported to be causative variants for gene expression change. A previous study by the 1000 Genomes Project reported that 0.56% (54/9591) of detected eQTLs had SVs as the leading variant [9], with 121 deletions reported as the lead variant. Another study also reported that SVs are causal for gene expression change for an estimated 3.5–6.8% of detected eQTLs, with 510 deletions being reported as the lead variant [17]. These studies, however, did not ascertain the accuracy of the identified deletions on a large scale. Furthermore, the studies were limited to European populations, and analysis of the impact of intermediate-sized deletions on gene expression has yet to be conducted for an Asian population. We therefore sought to identify intermediate-sized deletions in a Japanese population, determine their biological impact, and develop a method for a population study.

In the current study, we generated a catalog of intermediate-sized deletions from a whole-genome sequencing (WGS) cohort of 174 Japanese individuals and achieved highly accurate deletion detection by applying a joint-call recovery method. We then constructed a reference panel consisting of intermediate-sized deletions and SNVs for imputation. The accuracy of our analysis was evaluated at whole genome level using a long-read sequencing technology. Using the accurate list of intermediate-sized deletions, we also performed expression quantitative trait loci (eQTL) analysis with publically available datasets to analyze the functional impact of intermediate-sized deletions. We found that approximately 4.1% (181 out of 4378) of polymorphic intermediate-sized deletions were suggested to affect gene expression level. Our study showed a method for accurate intermediate-sized deletion calling, as well as indicating that these deletions have biological importance and analysis of intermediate-sized deletions can lead to the identification of novel disease susceptibility variants.

## Methods

### Samples selection

Whole-genome sequencing data from the ICGC Japanese samples were used in the current study [18]. We removed samples based on the presence of cryptic relationships, inappropriate proportions of homozygous and heterozygous SNVs, and analysis of population stratification by PCA (Additional file 1: Figure S1), resulting in 174 individuals for further study. This study was approved by the institutional review boards (IRBs) at RIKEN and all groups participating in this work.

### Detection of deletion candidates by IMSindel software

To identify deletions with high accuracy, we applied a joint-call recovery method and adapted a progressive deletion call for the first step. The IMSindel software (https://github.com/NCGG-MGC/IMSindel) [19] and output BAM files from the mapping of each of the 174 samples were used to identify deletions with the options “--indelsize 10000 --alt-read-depth 2 --support-reads 2.” Deletion candidates detected in each sample were combined and merged according to their breakpoint positions to generate an overall deletion candidate list. Deletion candidates that were < 30 bp in length were removed.

### Annotation and filtering of deletion candidates

Identified deletions were annotated according to their positions relative to (i) Refseq genes, (ii) telomeric or centromeric regions of chromosomes, (iii) simple repeats in the genome, and (iv) repeat-masked regions. Deletion candidates located in or overlapped by telomeric and centromeric regions, simple repeats, or microsatellites were excluded from further analysis. Deletion candidates with flanking regions (100 bp adjacent) of breakpoints within or overlapping simple repeats were also excluded. We also filtered out deletion candidates which had read depths over 150 or had higher read depths in the deletion region compared to the flanking regions, as were candidates with low average quality scores (< 15) of soft-clipped bases in the flanking regions of deletion breakpoints (Additional file 2: Supplementary note).

To further improve the accuracy of the detected deletion candidates and reduce the false-positive rate, we excluded deletion candidates having their breakpoints located within repetitive regions that were highly similar (e.g., two *Alu* transposons of the same class). The filtering process may remove deletion candidates in repetitive regions but improves specificity and ensures that the detected deletion candidates were of high quality and appropriate for use in imputation for association studies.

### Joint-call recovery of deletion candidates

IMSindel makes use of the information and quality of soft-clipped mapping reads to call deletions [19]. The sensitivity of variant calling in different samples can be influenced by read depth and quality, which in turn may be affected by local GC contents and random fluctuation. Typically, deletions called in the regions with a higher number of support reads or higher quality can be considered to be of higher confidence. With the IMSindel software, deletion calls are categorized according to those detected by soft-clipped reads in the forward-oriented reads (forward-type), reverse-oriented reads (reverse-type), or by both types. An increased read depth or quality is likely to result in deletion calls supported by forward and reverse reads. Reduced read depth or quality can lead to deletion calls using only either forward- or reverse-type reads and be of lower confidence. To improve the sensitivity of the deletion detected, we developed a joint-call method to retain the deletion calls within samples which may have deletions called from lower read depths, or only forward-type or reverse-type reads. The joint-calling considers deletions detected in multiple samples and retains deletions called using only forward-type or reverse-type reads in certain samples, if two or more other samples have the same deletion detected using both types of reads (Additional file 2: Supplementary note).

### Long-read sequencing technology for evaluation of detection accuracy

As long-read sequencing technology is expected to have technological advantages for SV calling compared to current short-read sequencing technologies [20], we leveraged it to assess the accuracy of IMSindel deletion calls. We sequenced whole genomes of two Japanese samples, RK067 and NA18943, using the Oxford Nanopore long-read sequencer and checked for the presence of the imputation results in the Nanopore dataset (Additional file 2: Supplementary note). We estimated the effectiveness and accuracy of the various filtering criteria, as well as the joint-calling method by comparing the deletion candidates against deletion calls from the Nanopore sequencing of sample RK067 (RIKEN ICGC sample) [18]. The results of imputation were compared against the deletion calls from the Nanopore sequencing of sample NA18943 (1000 Genomes sample) [21].

Library construction was done with SQK-LSK108 library construction kit (Oxford Nanopore) according to the manufacturer’s instructions. We performed 10 runs for RK067 and 19 runs for NA18943 with SpotON Flow Cell MK I (R9.4) (Oxford Nanopore). We mapped the reads to the human reference genome sequence with minimap2 software [22]. Since the error rates of Nanopore sequencing technology are known to be high [23], and that small-sized SVs are difficult to identify from Nanopore sequencing data due to the high error rate [24], we focused on identifying deletions of sizes ≥ 120 bp and supported by ≥ 2 reads with our own script. The analysis identified 34,624 deletions ≥ 120 bp in length, which were used for the evaluation. We considered deletions identified by Nanopore and the analysis of the short-read sequencing data as true positives, and the consistency rate between the two sets was evaluated.

### Intermediate-sized deletion imputation reference panel and imputation into genotype data

To assess the feasibility of using the deletion candidates for association/population studies, a reference panel for imputation was created. Data of biallelic variants (SNV and short indel) with minor allele frequency (MAF) of 5% and Hardy-Weinberg Equilibrium (HWE) *p* values > 0.0001 from the previous Japanese WGS study [18], with intermediate-sized deletion candidates filtered for HWE *p* values > 0.0001 (Fisher’s exact test), were combined to form the imputation reference panel (Additional file 2: Supplementary note). This reference panel was then used to impute the deletion candidates into genomic data of 82 Japanese samples from the 1000 Genomes Project database [21].

Genotype data of SNPs from the 82 Japanese samples used from the 1000 Genomes Project database [21] was obtained. The SNPs were filtered for those with MAF ≥ 0.05 and HWE *p* values > 0.00001. Imputation of the reference panel data into the 1000 Genomes dataset was conducted using IMPUTE2 [25] (http://mathgen.stats.ox.ac.uk/impute/impute_v2.html) (Additional file 2: Supplementary note). The effectiveness and accuracy of the imputation results were estimated by checking for consistency with the presence in deletion calls from Nanopore long-read sequencing data (Additional file 2: Supplementary note).

For the experimental validation of the accuracy imputation, we selected 11 deletion candidates (Additional file 3: Table S1) for 30 1000 Genomes JPT individuals, which were checked by PCR and gel electrophoresis. Primer sequences are provided in Additional file 3: Table S1.

### Gene expression association analysis and estimation of causative deletion candidates

To evaluate the potential functional impacts of the detected deletions, gene expression data for the 82 Japanese samples [26] was obtained from the EMBL-EBI ArrayExpress database and used for eQTL mapping with the imputation results. The eQTL mapping was conducted using the MatrixEQTL R package [27] (http://www.bios.unc.edu/research/genomic_software/Matrix_eQTL/), with the *cis*-window defined as 1 Mb from the transcription start site (TSS) of each gene on either side. Multiple testing correction was performed for each *cis*-window using the Benjamini-Hochberg (BH) method at 1% FDR for the imputed deletion candidates. The –log of observed association *p* values was plotted against those of expected association *p* values in a quantile-quantile (QQ) plot, and the genomic inflation value (*λ*) was calculated using R (ver. 3.3.3) for deletions with frequencies ≥ 5% in the Japanese HapMap samples. The CAVIAR software [28] (http://genetics.cs.ucla.edu/caviar/) was used to estimate if the imputed deletion candidates were causal for the changes in gene expression levels. Deletion candidates were considered as causal if they were contained within the set of suggested causal variants output by CAVIAR.

We also sought to determine if the causal deletion candidates are linked to significantly associated variants in previous disease GWAS. SNPs that were significantly associated (*p* value ≤ 5.0 × 10^−8^) in previously reported GWAS were obtained from the NHGRI-EBI Catalog of published genome-wide association studies [29], and the presence of these SNPs were checked in the CAVIAR causal set lists. If an associated SNP was present within a causal set list, the associated candidate deletion for that particular eQTL was considered to be linked with that particular GWAS-associated SNP.

### Annotation of regulatory features and enrichment analyses

We annotated the deletion candidates with regulatory features including transcription factor binding sites, super-enhancer sites, promoters and enhancers, CTCF binding sites, microRNA (miRNA) binding sites, and predicted chromatin states that the deletion candidates overlap or were located in (Additional file 2: Supplementary note). Additionally, the annotation was also done for the regulatory regions and features specific to the GM12878 B-lymphocyte cell line, which is a cell type used in the previous gene expression study [26]. To determine whether there was an enrichment of regulatory features for causal deletion candidates, counts of each regulatory feature were obtained for causal deletion candidates and compared to those of other deletion candidates using Fisher’s exact test. We further subdivided the causal deletion candidates into two categories: (i) deletion candidates causing gene expression increase and (ii) deletion candidates causing gene expression decrease, and the regulatory features of the two categories were also compared using Fisher’s exact test. Furthermore, we tested for enrichment of *Alu* transposons among the causal deletion candidates compared to non-causal deletion candidates. We considered the deletion candidates that overlapped at least 90% of an *Alu* transposon annotated region as *Alu* transposon deletions and thus focused on the deletion candidates of sizes 300–400 bp as they met this criterion. We defined other deletion candidates that did not meet the criterion as non-*Alu* transposon deletions. The enrichment analysis was conducted using Fisher’s exact test.

The average genome conservation scores were calculated for the deletion candidates using conservation scores of 45 vertebrate genomes obtained from the UCSC genome browser [30], compared to the human genome. The average conservation scores for casual deletion candidates were compared with those of other deletions, using the Wilcoxon rank-sum test.

### Phylogenetic status of deletion candidates

We furthermore classified the deletion candidates as ancestral or derived by comparing against the same region within the chimpanzee genome. A deletion candidate was considered “ancestral” if it was also present in the chimpanzee genome and “derived” if not seen in the chimpanzee genome (Additional file 2: Supplementary note). Enrichment analysis of phylogenetic statuses between causal and non-causal deletion candidates was conducted using Fisher’s exact tests.

### Generation of deletion with CRISPR-Cas9 system and examination of gene expression levels

To validate the effect of deletions on gene expression, we generated deletions in HEK293T cell lines (RIKEN Cell Bank) using the Alt-R CRISPR-Cas9 System (Integrated DNA Technologies). We aimed to generate a deletion (chr9:130330770-130330813), which was significantly associated with expression level change of the *FAM129B* gene, as well as another deletion (chr12:122230008-122230060), which was significantly associated with expression level change of the *TMEM120B* gene (Additional file 3: Table S2). Two gRNAs were designed to generate the deletion with sequences ATCCCAAAGCTGGTAGCGGATGG and ACTCGGAACTCCTTCTCTCCCGG for the deletion at chr9:130330770-130330813, while the gRNAs for the chr12:122230008-122230060 deletion had the sequences GTCCTCTCCAAGGTCTAGGTGTTTTAGAGCTATGCT and ACCTGCTGAAG TCGGAATGGGTTTTAGAGCTATGCT. Ribonucleoprotein complexes were generated for these gRNAs and transfected into HEK293T cells according to the manufacturer’s instructions. HEK293T cells were cultured in DMEM (Nacalai) supplemented with 10% FBS and antibiotics. Forty-eight hours after transfection, cells were diluted into a 96-well plate or 24-well plate at 1 cell/well. Wells with single cell clones were selected, and DNA was extracted 2–3 weeks after the dilution. Clones with and without the deletion were selected based on PCR and gel electrophoresis. DNA and RNA were extracted from the cells with QIAamp DNA Mini Kit (QIAGEN) and TRIzol reagent (Thermo Fisher Scientific) after approximately one week.

We estimated the expression level of *FAM129B* and *TMEM120B* by RT-PCR quantification, with *GAPDH* chosen as the reference gene. The primer sequences were AACAGCGACACCCATCCTC and CATACCAGGAAATGAGCTTGACAA for *GAPDH* [31], GGCTGGTGCTCTACGAAAACA and CACGGACGTGAGGATTTTGTA for *FAM129B*, and CTTACACTCCAGAGGTGCAAAC and CGCTCCTTGATGTTCGCTG for *TMEM120B*. After DNase I treatment (Invitrogen), total RNA was reverse-transcribed with PrimeScript RT reagent kit (Takara). The cDNA was used for qPCR with TB Green Premix Ex Taq II (Takara) according to the manufacturer’s instructions using a StepOnePlus Real-Time PCR System (Thermo Fisher Scientific). The real-time PCR reaction for each sample was run in triplicate, and the average relative quantification (RQ) values were obtained. The statistical significance of RQ values between the clones with and without deletions was performed using the Wilcoxon rank-sum test.

## Results

### Detection of intermediate-sized deletion candidates using filtering and joint-call approach

**Fig. 1:**
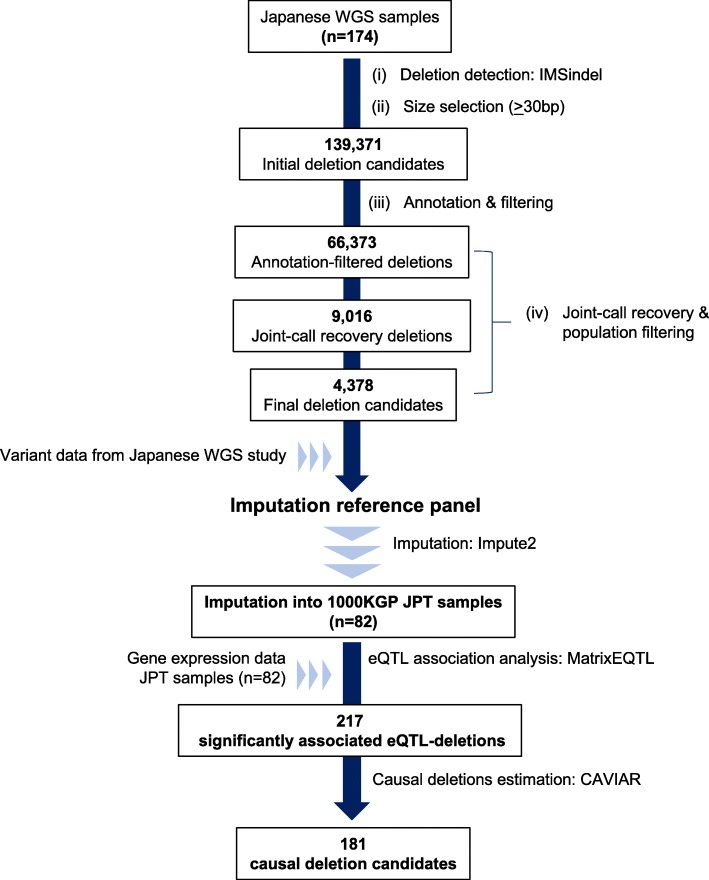
Workflow of the current study. The figure briefly describes the workflow of the current study. From 174 Japanese WGS samples, an accurate list of intermediate-sized deletions was identified after application of filtering and joint-call recovery methods. An imputation panel was generated and these deletions imputed into a separate set of Japanese genomic data. Deletions that were estimated to cause gene expression level changes were then identified after an eQTL association analysis

**Fig. 2:**
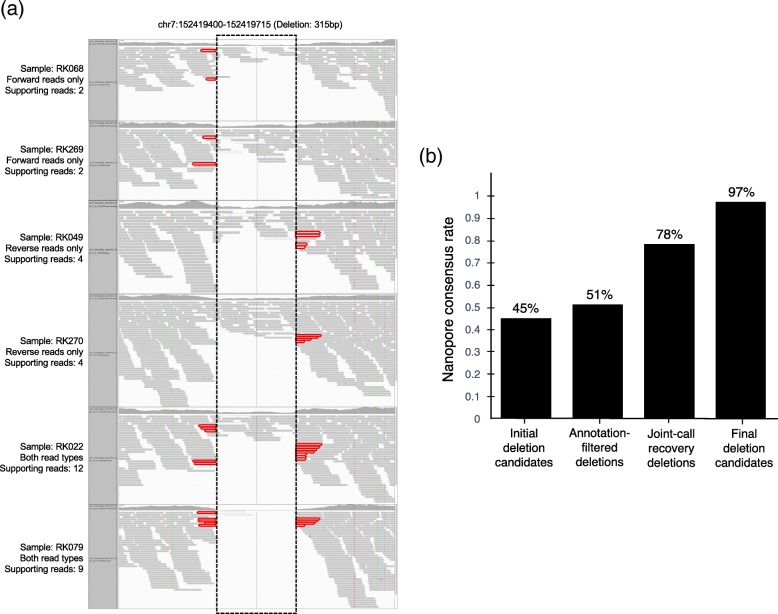
Example of joint-call recovery of deletion candidate and effectiveness of progressive filtering procedure. **a** Example of a deletion (chr7:152419400-152419715; 315 bp deletion) that was identified after applying the joint-call recovery. The deletion region is enclosed by the dashed box, and sequencing reads used by IMSindel to detect the deletion’s breakpoints are highlighted in red. For the first four samples, the deletion was detected by IMSindel using only forward or reverse reads, while both read types were used for the detection in the following two samples, leading to a higher confidence deletion call. **b** The true-positive rate estimation of detected deletion candidates at each processing step by comparison with deletions detected by Nanopore long-read sequencing. The processing was effective in improving the accuracy of deletion calls, with only a 45.2% consensus rate of the initial detected deletion candidates by IMSindel. Improvement in consensus rate was seen at each processing step, with a final consensus rate of 97.3% seen for the high-confidence deletion candidates

From the WGS data of Japanese samples [18], we removed samples based on population filters, resulting in 174 samples remaining for analysis. Deletions were called using the IMSindel software [19], and after selecting for deletion candidates ≥ 30 bp, a total of 139,371 deletion candidates were detected by IMSindel (Fig. 1). We applied a filtering and joint-call recovery method to obtain high-confidence deletion candidates (Fig. 2a), with 66,373 deletions remaining after filtering (Fig. 1). After joint-call recovery and population filtering of deletion candidates with Hardy-Weinberg Equilibrium (HWE) *p* value < 0.0001, a final 4378 deletion candidates remained for further analysis (Fig. 1 and Additional file 3: Table S3).

### Evaluation of deletion detection accuracy using long-read sequencing technology

To assess the accuracy of the deletion calls by IMSindel and the genomic imputation, the whole genome of a sample that was used in the previous Japanese WGS (RK067) [18], as well as the current study, was sequenced by Oxford Nanopore long-read sequencer. We detected deletions ≥ 120bp in length from the long-read sequencing data, which we considered as true positives. These were compared against IMSindel deletion calls to estimate the consistency rate of each processing step in the filtering and joint-call recovery of deletion candidates (Additional file 2: Supplementary note). The true-positive rate for overall detected deletion candidates prior to filtering was determined to be 45.2% (1610/3563 deletions) due to the progressive calling parameter set, with each processing step resulting in an increase in the true-positive rate. The final true-positive rate for high-confidence deletion candidates was seen to be 97.0% (928/958 deletions) (Fig. 2b). This large-scale comparison indicates that the accuracy of our deletion calling methods was high.

### Features of deletions

**Fig. 3:**
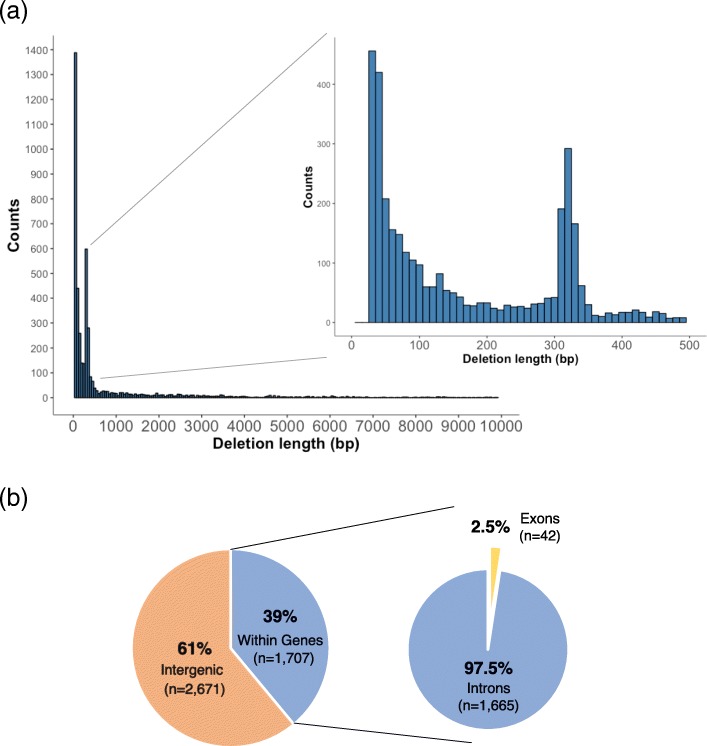
Distribution of intermediate-sized deletions within the genome. **a** The histogram shows the size distribution of detected high-confidence deletion candidates. The majority of deletion candidates were seen to be of lengths shorter than 1 kbp. Two distinct peaks were observed, the first for deletion candidates of sizes between 30 and 100 bp and the second peak for deletion candidates of sizes between 300 and 400 bp, which are likely representative of the presence of SINE *Alu* transposons in the genome. **b** The locations in which deletion candidates were located in. The majority (61.0%) of deletion candidates were located in the intergenic regions, while 39.0% were located within genes. Of those within genes, only a small fraction (2.5%) were seen to be within or overlapping the exons, while the majority (97.5%) were within the intronic regions

Among the deletion candidates, the majority were seen to be between 30 and 100 bp in size (Fig. 3a). Deletion candidates of sizes between 300 and 400 bp made up the second most abundant category of deletion candidates (Fig. 3a), and a substantial majority (87.3%) were seen to contain or intersect an *Alu* transposon (Additional file 3: Table S2). The majority of deletion candidates (61.0%) were also observed to be located within the intergenic regions (2671/4378), while 39.0% (1707/4378) were located in genes, with 1.2% (20/4378) of these deletions seen to be located in the UTR regions of genes (Fig. 3b and Additional file 3: Table S3). Of the deletion candidates located in genes, only a small fraction (2.5%; 42/1707) were observed to be located in or overlapping the exon regions (Fig. 3b and Additional file 3: Table S3), indicating that these deletion candidates could likely be gene-disrupting and possibly of functional importance. It is also possible that selection pressure acts against and removes deletions that overlap exons.

### Imputation of deletion candidates for Japanese 1000 Genomes samples and validation of deletion candidates

**Fig. 4:**
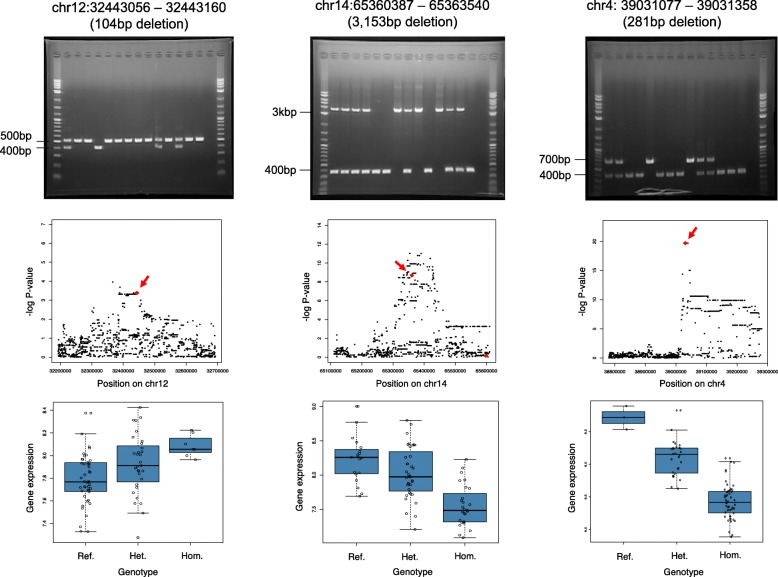
Validation of imputed intermediate-sized deletions by PCR and association with gene expression. Electrophoresis results of the PCR validation for three of the eleven imputed deletion candidates that were selected for validation. High concordance between the PCR validation and imputation results were observed (Additional file 3: Table S3). The eQTL association *p* value plots of the deletion candidates are shown below the electrophoresis results. The red arrows indicate the locations of the deletion candidates (red diamonds). The boxplots below show the change in gene expression levels brought about by the deletion candidates

A reference panel for imputation was created using SNVs [18] and the dataset of deletion candidates in the current study (see the “Methods” section and Additional file 2: Supplementary note). The combination of SNVs [18] and deletion candidates resulted in a reference imputation panel consisting of 5,244,299 variants. The reference panel was used for imputation into genotype data of 82 Japanese samples from the 1000 Genomes Project database [21], and the imputation resulted in a total of 421,690 variants (SNVs and deletions) being imputed into the Japanese samples. At an imputation genotype probability threshold of 0.5, all deletion candidates (*n* = 4378) were successfully imputed. To estimate the accuracy of the imputation, we also sequenced the whole genome of a 1000 Genomes JPT sample (NA18943) [21] using Nanopore sequencing and compared the deletions ≥ 120 bp called with the imputation results. The comparison found that 97.3% (921/947) of polymorphic deletions were accurately imputed, while 98.3% (1514/1540) of reference alleles were correctly imputed. The 2.7% of inconsistently imputed deletions were observed to have lower frequencies of individuals with the deletion (Additional file 1: Figure S2). We further validated the accuracy of the imputation by selecting 11 deletion candidates and conducting PCR for them in 30 1000 Genomes JPT individuals (see the “Methods” section). The PCR validation of deletion candidates also showed that the imputation results were accurate, with a 98.2% consensus rate between the imputed results and PCR results (Fig. 4 and Additional file 3: Table S1).

### Associated eQTLs and causal deletion candidates

The results of the imputation were then used for eQTL mapping with gene expression data of the 1000 Genomes Japanese samples obtained from a publically available dataset [26] (see the “Methods” section). No genomic inflation was observed for the eQTL association results, with a genomic inflation value (*λ*) of 0.96 (Additional file 1: Figure S3), suggesting that there was no systematic inflation of the test statistics. We also estimated which deletion candidates were causative for the gene expression level changes using the CAVIAR software, which estimates a set of potentially causal variants for associated loci by considering the correlations (e.g., LD structure) between variants within a set region around the association loci [28]. We identified 217 significantly associated unique eQTL deletions at *q* value ≤ 0.01. Of these 181 deletion candidates (83.4%) were suggested to possibly be causal by CAVIAR software (Additional file 3: Table S2).

Additionally, we checked if the causal deletion candidates were in linkage disequilibrium to disease-associated variants reported by previous GWAS studies (see the “Methods” section). We observed that 13.3% (24/181) of the causal deletion candidates were linked to disease-associated variants, including diseases such as systemic lupus erythematosus (SLE), atrial fibrillation, schizophrenia, psoriasis, selective IgA deficiency, and Hirschsprung disease, among others (Additional file 3: Table S2).

### Enrichment of regulatory features in causal deletion candidates

**Fig. 5:**
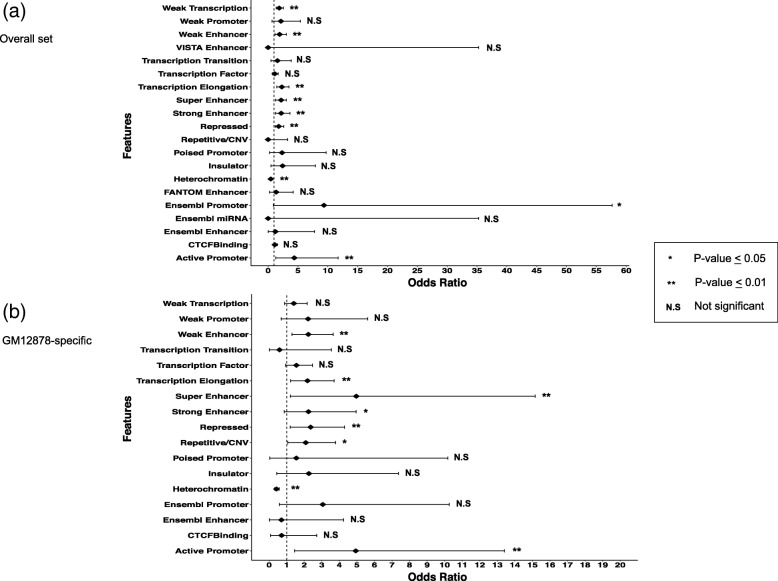
Enrichment of annotated regulatory features. **a** Odds ratios (ORs) for significantly enriched regulatory features (overall set) between suggested causal deletion candidates and other deletion candidates. The largest effect was seen for Ensembl promoters (OR = 9.35) while chromatin states such as heterochromatin showed a negative enrichment effect (OR = 0.45). **b** ORs for significantly enriched GM12878-specific regulatory features between suggested causal deletion candidates and other deletion candidates. The largest effect was seen for the super-enhancers (OR = 4.97), with the active promoter predicted chromatin state also showing large effect (OR = 4.94). Negative enrichment effect was seen for the heterochromatin state (OR = 0.42)

Of the 181 causal deletion candidates, 93 (51.4%) were observed to be located within genes, with 4 deletion candidates (2.2%) seen to be located in gene exons and the majority of deletion candidates were seen to locate in non-genic regions (Additional file 3: Table S2). Therefore, we sought to examine the importance of regulatory regions in terms of affecting gene expression. To that end, we further annotated the deletion candidates with regulatory features from publically available databases (see the “Methods” section and Additional file 2: Supplementary note) and compared the proportion of deletions with each feature between the 181 causal deletions and others. Enrichment of certain regulatory features was observed between causal deletion candidates and other deletion candidates (Additional file 3: Table S4). Interestingly, super-enhancers were seen to be highly enriched (*p* value = 8.6 × 10^−6^; OR = 2.18) in suggested causal deletion candidates, and enrichment was also seen for 7 predicted chromatin states, such as transcription elongation (*p* value = 2.3 × 10^−4^; OR = 2.30), weak transcription (*p* value = 8.7 × 10^−5^; OR = 1.86), active promoters (*p* value = 0.0093; OR = 4.38), and strong enhancers (*p* value = 0.0045; OR = 2.19) (Fig. 5a and Additional file 3: Table S4). In contrast, the opposite effect was seen for heterochromatin (*p* value = 8.0 × 10^−5^; OR = 0.45) (Fig. 5a and Additional file 3: Table S4).

For the regulatory features specific to the GM12878 B-lymphocyte cell line, we observed that of the 181 causal deletion candidates, 4 deletion candidates (2.2%) were seen to overlap or locate within or partially within the super-enhancer regions, and 70 deletion candidates (39.0%) overlapped or were located in the CTCF binding sites (Additional file 3: Table S2). Furthermore, 22 deletion candidates (12.2%) overlapped or partially covered the transcription factor binding sites (Additional file 3: Table S2). Enrichment was observed for super-enhancers (*p* value = 0.014; OR = 4.96) and the chromatin states of transcription elongation (*p* value = 0.0061; OR = 2.19), repressed chromatin (*p* value = 0.0087; OR = 2.36), active promoters (*p* value = 0.0060; OR = 4.94), and strong enhancers (*p* value = 0.049; OR = 2.24) (Fig. 5b). An opposite effect was again observed for the heterochromatin state (*p* value = 5.9 × 10^−8^; OR = 0.42) (Fig. 5b and Additional file 3: Table S5). We compared the proportion of deletions with each feature between the gene expression decreasing deletions and increasing deletions; however, no difference was seen (Additional file 3: Table S6).

Next, we focused on deletions overlapped with *Alu* transposons and average conservation score of deletions. The *Alu* transposon deletions were found in the causal deletions and showed both increasing and decreasing effects on gene expression levels (Additional file 1: Figure S4). This result suggests that deletion of *Alu* transposons can cause regulatory changes in gene expression. No enrichment of *Alu* transposon was seen when causal deletion candidates were compared against non-causal deletion candidates (Additional file 3: Table S7). The comparison of average genomic conservation score between the causal and non-causal deletions showed that causal deletions had significantly lower conservation scores; however, the difference was not large (Wilcoxon rank-sum test *p* value = 0.018, Additional file 1: Figure S5). We also compared the size of deletions between the two categories, but no significant difference was observed (Additional file 1: Figure S6).

### Phylogenetic status of causative deletion candidates

Within the 4378 deletion candidates, the phylogenetic status of 4152 were obtained, and of these, 22.5% were found in the chimpanzee genome and considered as ancestral. Derived deletions were found only in the human genome and should be generated by deletion events within the human population. In contrast, ancestral deletions are shared with chimpanzees. It is plausible that they are caused by insertion events occurring within the human population, resulting in the human reference genome also having the insertion. Since our analysis detected deletions based on the comparisons with the human reference genome, these events are detected as deletions.

Enrichment analysis was conducted for causative deletion candidates and it was observed that the proportion of ancestral deletions was higher than that of derived deletions (Fisher’s exact test *p* value = 0.075 [non-significant]; OR = 1.37). Additionally, we found that deletions overlapping *Alu* transposons were significantly overrepresented in the ancestral deletion category (*p* value < 1.0 × 10^−180^; OR = 460.03) (Additional file 3: Table S8), and this suggests that a large number of polymorphic *Alu* insertion events exist within the human reference genome. Derived deletion candidates were also observed on average to have significantly lower frequencies of individuals with the deletion compared to ancestral deletion candidates (Wilcoxon rank-sum test *p* value = 1.9 × 10^−180^, Additional file 1: Figure S7).

### Generation of deletion with CRISPR-Cas9 system and examination of gene expression levels

**Fig. 6:**
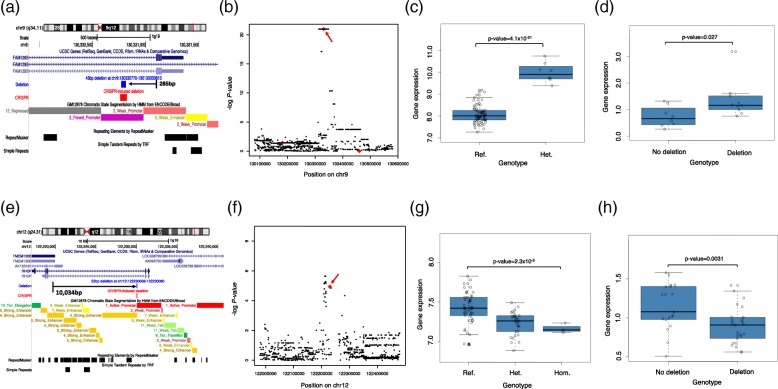
Results of deletions generation in HEK293T cells and effects on gene expression. **a** Location of 43 bp deletion at chr9:130330770-130330813 and annotation of the region. The deletion is located in an intronic region on the *FAM129B* gene, approximately 285 bp away from the nearest exon. The deletion is indicated by the blue bar while the red bar below shows the deletion induced by the CRISPR-Cas9 system. The purple bar indicates the region annotated as having a poised promoter chromatin state according to the ENCODE/Broad database. **b** Result of eQTL analysis. The eQTL association *p* value plot for deletion is shown. The red arrow indicates the location of the deletion at chr9:130330770-130330813 (red diamond). The deletion is seen to be a top eQTL hit within the region. **c** Boxplot of gene expression in the eQTL analysis. The boxplot shows the result of gene expression level change in the eQTL association analysis for the deletion at chr9:130330770-130330813. **d** Comparison of gene expression levels between HEK293T clones with and without the chr9:130330770-130770813 deletion. The *y*-axis shows the average relative quantification (RQ) values of the qPCR triplicate experiment. Significant differences were seen for gene expression levels between clones with and without the deletion (Wilcoxon rank-sum test *p* value = 0.027). **e** Location of 52 bp deletion at chr12:122230008-122230060 and annotation of the region. The deletion is located in an intronic region of the *RHOF* gene, approximately 10 kb away from the nearest exon of the *TMEM120B* gene which gene expression level is affected. The deletion is indicated in blue while the CRISPR-Cas9-induced deletion is shown in red. **f** Result of eQTL analysis. The eQTL association *p* value plot for the deletion is shown. The red arrow indicates the location of the deletion at chr12:122230008-122230060 (red diamond). The deletion is seen to be one of the top association hits in the region. **g** Boxplot of gene expression of the eQTL analysis. The boxplot shows the result of gene expression level change in the eQTL analysis for the deletion at chr12:122230008-122230060. **h** Comparison of gene expression levels between HEK293T clones with and without the chr12:122230008-122230060 deletion. The *y*-axis shows the average relative quantification (RQ) values of the qPCR triplicate experiment. Significant differences were seen for gene expression levels between clones with and without the deletion (Wilcoxon rank-sum test *p* value = 0.003)

Our analysis showed that the causal deletion candidates are enriched in several functional categories, suggesting that the majority of them are true causative variants. To prove the functional impact of deletions directly, we induced deletions into HEK293T cells, and examined the effect on gene expression level using quantitative real-time RT-PCR. We chose the deletions chr9:130330770-130330813 and chr12:122230008-122230060 for the experiment (Fig. 6a, e). Using the CRISPR-Cas9 system, we induced deletions into HEK293T cell lines (Fig. 6a, e; Additional file 2: Figures S8 and S9). We obtained 10 and 8 clones with and without the deletion, respectively, for deletion chr9:130330770-130330813, as well as 25 and 19 clones with and without the deletion, respectively, for deletion chr12:122230008-122230060. The qPCR experiment showed that clones with the deletion had higher gene expression levels as suggested by the eQTL analysis for chr9:130330770-130330813 (Wilcoxon rank-sum test *p* value = 0.027) (Fig. 6b–d). For chr12:122230008-122230060, clones with the deletion had lower gene expression levels as suggested by the eQTL analysis (Wilcoxon rank-sum test *p* value = 0.003) (Fig. 6f–h). This result strongly suggests that the deletions are causal, and deletions in intronic regions can have a functional effect on gene expression.

## Discussion

The importance of SVs and small indels has been shown in their associations with numerous diseases [5–8], and intermediate-sized deletions can also be expected to have important impacts in genome structure or have important functional impacts. Results from earlier GWAS for disease and/or trait associations have shown that a majority of the association signals lie outside of gene coding regions [3, 4], and such intermediate-sized deletions may represent an important aspect to explain the missing heritability seen thus far with GWAS. Systemic identification and analysis of such intermediate-sized deletions have thus far been limited by the difficulty in accurately detecting them. In the current study, we successfully identified a set of accurate, high-quality intermediate-sized deletions from WGS data of 174 Japanese samples, by developing a filtering and joint-call recovery pipeline. Previous studies that have been conducted that analyzed SVs at a genome-wide level have been limited to European populations [9, 17]. Our current study is the first to provide a systematic evaluation of SV call accuracy using a long-read sequencing technology, as well as the first to evaluate the effects of such SVs on gene expression in an Asian population. Furthermore, we successfully generated a reference panel and showed that these deletions can be accurately imputed into the genome for further application to population or disease-association studies. We further analyzed the relation of these deletion candidates with gene expression level changes and found that approximately 4.1% of the deletion candidates were suggested to be the causal variants for such changes. We also revealed the features of causal deletion candidates, and this can contribute to identification of functional deletions. Our study additionally found that of the 181 causal deletion candidates, 24 (13.3%) were in strong LD with SNPs that were previously reported to be significantly associated with diseases/phenotypes GWAS, suggesting that these deletion candidates can be considered as more likely to be the causal variants rather than the GWAS-associated SNPs. It would be worthwhile to test such intermediate-sized deletions with association to other diseases or phenotypes.

For identifying genetic variations, development of variant calling methods and evaluation of false positive and negative rates are very important. Generally, there is a trade-off between sensitivity and specificity; progressive thresholds cause higher sensitivity but lower specificity, and vice versa. To achieve higher sensitivity and specificity, we detected deletions with a progressive threshold and then applied a filtering and a joint-call recovery method. The joint-calling leveraged on the presence of deletions in the same location that were detected in different samples. Higher-confidence deletion calls were used to recover lower-confidence deletion calls in other samples, thereby leading to a reduction in loss of detection sensitivity. To evaluate the accuracy of this approach, we used a long-read sequencing technology and found that the consistency with the long-read sequencing technology was sufficient for intermediate-sized deletion cataloging and imputation (deletion detection (RK067), 97.0%; imputation (NA18943), 97.3%; and population frequency of imputation (PCR of 30 1000 Genomes samples), 98.2%). This is comparable with a previous Dutch trio study (98%) [32] and higher than another previous 1000 Genomes Project European population study (71.6%) [33]. Although there are 2.7% of inconsistent deletions, frequencies of individuals with the deletions were lower than others (Additional file 1: Figure S1), suggesting that a larger sample size study can reduce the inconsistency rate. This comparison allowed us a degree of confidence in the accuracy of the detected deletion candidates and the created reference panel. To our knowledge, a suitable method for assessing the accuracy of deletion/SV calls from short-read sequencing data has yet to be developed, and our study is the first to describe such an approach.

We analyzed the association between the deletion genotypes and gene expression levels (eQTL analysis) following the high-quality imputation. The eQTL analysis identified 217 deletion candidates that were associated with gene expression level changes, of which 83.4% (*n* = 181) of the candidates were suggested to be causal for the changes in gene expression levels. We observed relatively low numbers of causative deletion candidates located within gene exons, indicating that deletions that affect gene expression would be more likely to be found in regions related to regulation instead. Indeed, causative deletion candidates were significantly enriched in regulatory regions such as super-enhancer sites, regions of active promoter, strong enhancer, and transcription elongation chromatin states. In contrast, heterochromatin regions were significantly underrepresented in causative deletions. Heterochromatin regions are understood to be transcriptionally silent as they are relatively inaccessible regions in the genome [34]. The underrepresentation of causal deletions in such regions can be expected, given their transcriptionally silent nature. This suggests that deletions that were important in affecting gene expression levels were more likely to be located in the regions of the genome that were open for transcription, thus allowing for the changes in gene expression levels to be seen. Furthermore, we found that deletion size was not significantly different between the causative and non-causative deletions (Additional file 1: Figure S5). These results suggest that deletions in known regulatory regions have higher importance, regardless of size. Although we compared the features of deletions causing gene expression increase and decrease, no clear pattern emerged. It is likely that the functional impacts of causative deletions on gene expression levels are dependent on the genomic context of the region and that the mechanism of gene expression regulation is highly complex.

To directly prove the biological impact of deletions, we induced deletions (chr9:130330770-130330813 [rs531030622] and chr12:122230008-122230060) to HEK293T cells. The deletions were small (43 bp and 52 bp) and were located in the intronic regions. The deletion chr9:130330770-130330813 locates deep in an intron (285 bp from the nearest exon) (Fig. 6a) of the *FAM129B* gene, and the functional importance of this kind of deletion has hitherto not been well-studied. The experimental validation of this deletion showed that gene expression levels of this gene were indeed increased by the presence of the deletion (Fig. 6d), and we observed a large positive gene expression change effect. The deletion was also seen to be located within the binding site of the *SUZ12* polycomb repressive complex 2 subunit, which has been previously suggested to function as a gene silencer [35]. It is likely that the deletion within this region could affect the binding of *SUZ12* and reduced/removed the silencing function to cause an increase in gene expression levels. It is commonly thought that the deletions would always lead to decrease/disruption of gene expression levels, but our eQTL analysis and this experiment showed that deletions can also cause increasing gene expression levels. Additionally, the deletion chr12:122230008-122230060 is located in an intron of the *RHOF* gene, about 10,034 bp from the nearest exon of the *TMEM120B* gene, which the expression level is affected by the deletion (Fig. 6e). The experimental validation showed that as predicted in the eQTL analysis, the presence of the deletion caused a decrease in gene expression levels (Fig. 6h). Interestingly, the deletion is able to elicit gene expression level changes despite being located in the intronic region of another gene. The deletion was located within a super-enhancer region (Additional file 3: Table S2) and looping of such enhancer regions with target genes’ promoter regions [36] may allow the deletion to affect another gene’s expression level. The *RHOF* gene has also been suggested to have enhancer interaction with the *TMEM120B* gene by GeneHancer [37], which may explain how the deletion is able to affect the gene expression of the *TMEM120B* gene. Super-enhancer regions are also relatively large [36], of which the deletion takes up only a small portion. This could suggest that specific portions of super-enhancer regions are responsible for affecting the expression of different genes. Our analysis clearly shows that variants can affect the expression levels of genes they are not located in. Furthermore, the deletion was causal for gene expression level change although it was not the most significantly associated variant within the region when compared with SNVs (Fig. 6f). This suggests that deletions can have higher functional impact compared to SNVs. Thus, it would be worthwhile to also consider deletions when identifying causative variants, even if the deletions were not the most significantly associated.

The enrichment of ancestral deletions that were overlapped with *Alu* transposons was also rather interesting, as it indicated that it is likely these regions arose from insertion events in the human population before making their way into the human reference genome, rather than being truly deleted regions. Furthermore, the presence of such *Alu* transposon deletions that cause significant gene expression level changes (Additional file 1: Figure S3) might point to *Alu* transposons being not as neutral in their effects in the genome as previously thought [38]. Also, as observed with other deletions in regulatory features such as transcription factors, *Alu* transposon deletions can also affect the gene expression levels in both directions, further demonstrating that the regulation of gene expression is an extremely complex process.

Limitations do exist for the current study, however. In this study, we focused on intermediate-sized deletions, because identification of the entire structure of deletions can be observed using current short-read sequencing technologies, and it is not difficult to generate deletions by the CRISPR-Cas9 system. This would allow for a clearer understanding of the variants’ functions. A previous study showed that 49% of SVs were deletions [17], suggesting that analysis of intermediate-sized deletions can account for approximately half of the SVs in the human genome. Although identification of the entire structures and generation of other types of SVs, such as insertion and inversions, are difficult given current technologies, development of new algorithms and improvement of genome editing methods would enable us to detect them, as well as analyze their biological impacts. Additionally, we also removed deletions by repeat and HWE filtering. We considered that this was necessary to obtain a reliable deletion list, but this process would have also removed true deletions in repetitive regions. Indeed, in our analysis, we found that within the Nanopore-matched deletion candidates for RK067 sample, there was a loss of 682 deletion candidates between the overall and high-confidence deletion candidates’ datasets, due to the majority of the deletion candidates locating in such technically challenging regions. In the future, we expect that such issues may be resolved by long-read sequencing technologies such as Nanopore, with larger numbers of functional deletions being identified and used in population studies.

## Conclusions

The current study identified a set of high-confidence intermediate-sized deletion candidates from whole-genome sequencing of a Japanese population using a novel, multi-sample deletion calling method, and filtering process. A reference panel for genomic imputation was created from the detected deletions, and high-accuracy imputation was achieved. Subsequent eQTL association analysis identified deletion candidates that were causal for gene expression level changes and were seen to be enriched in important regulatory regions. Intermediate-sized deletions are therefore functionally important, and further disease/phenotype association studies should be conducted using these deletions.

## Additional files

Additional file 1: **Figure S1.** Principal component analysis (PCA) plot of the study samples with the HapMap samples. **Figure S2.** Boxplot showing the frequencies of individuals with deletion between consistently and inconsistently imputed deletions. **Figure S3.** Quantile-quantile plot of expected and observed eQTL association *p* values. **Figure S4.** Examples of association *p* value plots and gene expression boxplots for causal deletion candidates that overlapped Alu transposons. **Figure S5.** Boxplot of genome conservation scores between causal and non-causal deletion candidates. **Figure S6.** Boxplot showing the size distribution between causal and non-causal deletion candidates. **Figure S7.** Boxplot showing the frequencies of individuals with deletion between ancestral and derived deletion candidates. **Figure S8.** Gel electrophoresis of CRISPR-Cas9-induced deletions for a deletion at chr9:130330770-130330813. **Figure S9.** Gel electrophoresis of CRISPR-Cas9-induced deletions for a deletion at chr12:122230008-122230060. **Figure S10.** Manhattan plot of variants with intermediate-sized deletions highlighted. (PDF 2173 kb).

Additional file 2: Supplementary note. File containing supplementary information. (PDF 142 kb)

Additional file 3: **Table S1.** List of eQTL causal intermediate-sized deletions chosen for PCR validation, and the results of the PCR validation using 30 1000 Genomes JPT samples. **Table S2.** Annotation of regulatory features and predicted chromatin states for identified intermediate-sized deletions. **Table S3.** List of highconfidence intermediate-sized deletions after detection and filtering. **Table S4.** Results of enrichment analysis for annotated overall regulatory features. **Table S5.** Results of enrichment analysis for annotated GM12878 cell line-specific regulatory features. **Table S6.** Results of enrichment analysis of annotated regulatory features comparing causal intermediate-sized deletions that increased gene expression levels against those with decreased gene expression levels. **Table S7.** Results of enrichment analysis for deletion candidates overlapping Alu transposons. **Table S8.** Results of enrichment analysis for phylogenetic status of causal and non-causal deletions, as well as for deletions the overlap Alu transposons. (XLSX 5262 kb)

## Data Availability

The data used in the current study are as follows: Gene expression data for 82 Japanese HapMap samples used for the eQTL association analysis were taken from EMBL-EBI ArrayExpress under accession E-MTAB-264 [39]. Nanopore MinION long-read sequencing data for sample RK067 is available from the Japanese Genotype-phenotype Archive (JGA) database (https://ddbj.nig.ac.jp/jga/viewer/view/studies) under accession number JGAS00000000180. Nanopore MinION long-read sequencing data for sample NA18943 is available from the DNA Data Bank of Japan (DDBJ) Sequence Read Archive (DRA) (https://www.ddbj.nig.ac.jp/dra/index-e.html) under accession number DRA008482. Source code is available from https://github.com/JamesWongJingHao/intermediate-deletions-joint-call-recovery. The reference panel used for the imputation in the current study is not publicly available due to the risk of compromise of participant privacy but are available from the corresponding author on reasonable request. Published GWAS association results are available from NHGRI-EBI Catalog of published GWAS (https://www.ebi.ac.uk/gwas/home) [29]. Super-enhancer region data is available from dbSUPER (http://asntech.org/dbsuper/) [40]. CTCF binding site data is available from CTCFBSDB2.0 (http://insulatordb.uthsc.edu/) [41]. Ensembl regulatory region data was obtained using BioMart for Ensembl Release 93 (https://grch37.ensembl.org/info/index.html) [42]. UCSC phastCons genome conservation scores are available from the UCSC genome browser (http://hgdownload.cse.ucsc.edu/goldenpath/hg19/phastCons46way/) [43].
