# Supplementary material for: Identification of intermediate-sized deletions and inference of their impact on gene expression in a human population
Source: Genome Med. 2019 Jul 24;11:44. doi: 10.1186/s13073-019-0656-4 (PMC6657090; doi:10.1186/s13073-019-0656-4)
Supplement: Supplementary file 1 — Figure S1. Principal component analysis (PCA) plot of the study samples with the HapMap samples. Figure S2. Boxplot showing the frequencies of individuals with deletion between consistently and inconsistently imputed deletions. Figure S3. Quantile-quantile plot of expected and observed eQTL association p values. Figure S4. Examples of association p value plots and gene expression boxplots for causal deletion candidates that overlapped Alu transposons. Figure S5. Boxplot of genome conservation scores between causal and non-causal deletion candidates. Figure S6. Boxplot showing the size distribution between causal and non-causal deletion candidates. Figure S7. Boxplot showing the frequencies of individuals with deletion between ancestral and derived deletion candidates. Figure S8. Gel electrophoresis of CRISPR-Cas9-induced deletions for a deletion at chr9:130330770-130330813. Figure S9. Gel electrophoresis of CRISPR-Cas9-induced deletions for a deletion at chr12:122230008-122230060. Figure S10. Manhattan plot of variants with intermediate-sized deletions highlighted. (PDF 2173 kb). [file 13073_2019_656_MOESM1_ESM.pdf]

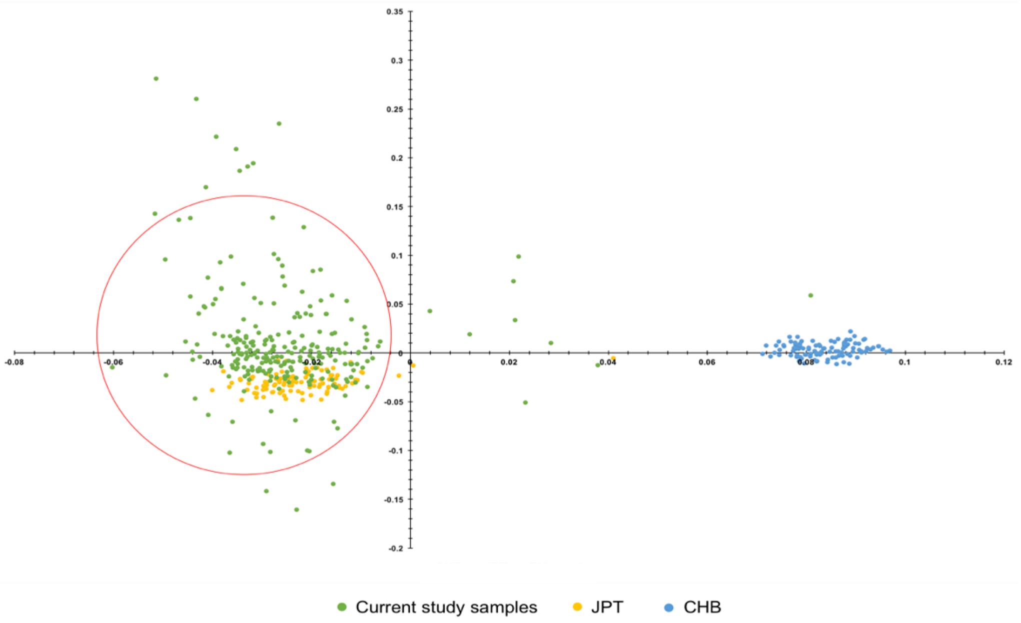

Figure S1: Principal Component Analysis (PCA) plot of the samples used in the current study with those of the HapMap Japanese (JPT) and Chinese (CHB) samples. Blue points on the plot represent CHB individuals, while yellow points represent JPT individuals. Current study samples are represented by green points. The red circle on the plot represents the cluster from which current study samples were taken for further analysis.

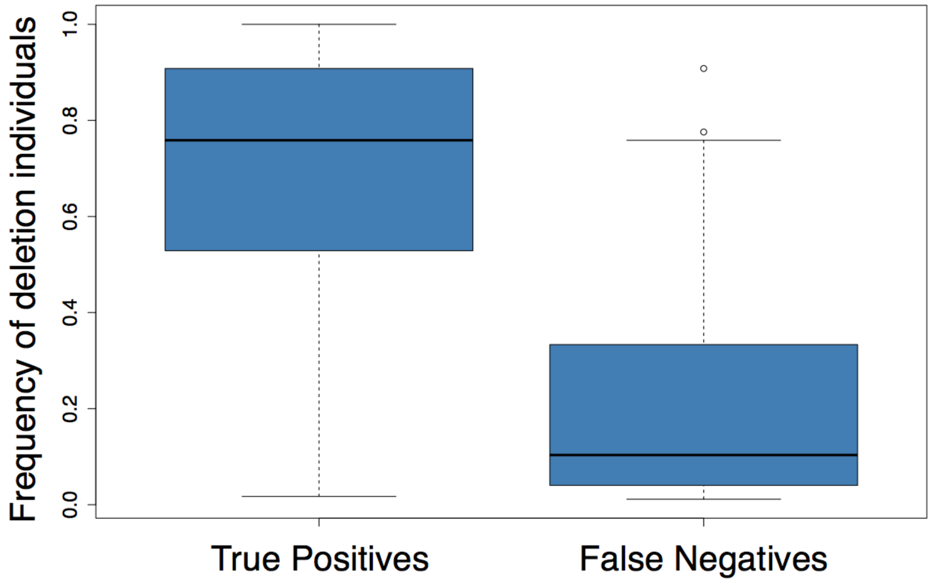

Figure S2: Lower frequency of individuals with deletions for inconsistently imputed deletions. The figure shows the plot of the frequency of individuals with deletions between the imputed intermediate-sized deletions that were observed to be consistent with Nanopore-detected deletions (True Positives) and those that were inconsistent (False Negatives). The inconsistent deletions were observed to have generally lower frequencies of individuals with deletions than those of consistent deletions.

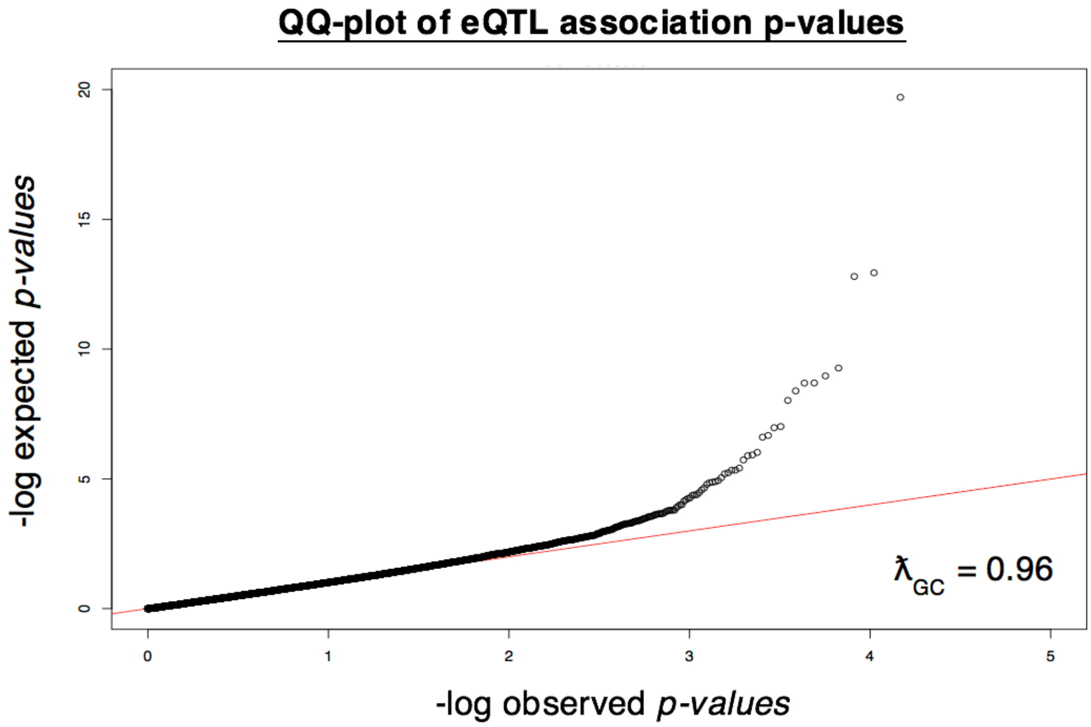

Figure S3: QQ-plot of expected and observed association p-values of eQTL analysis.

The quantile-quantile plot of the –log of expected association p-values and observed association p-values from the eQTL association analysis. The genomic inflation factor (λ_{GC}) was calculated to be 0.96, indicating that there was no systemic inflation of test statistics.

(a)

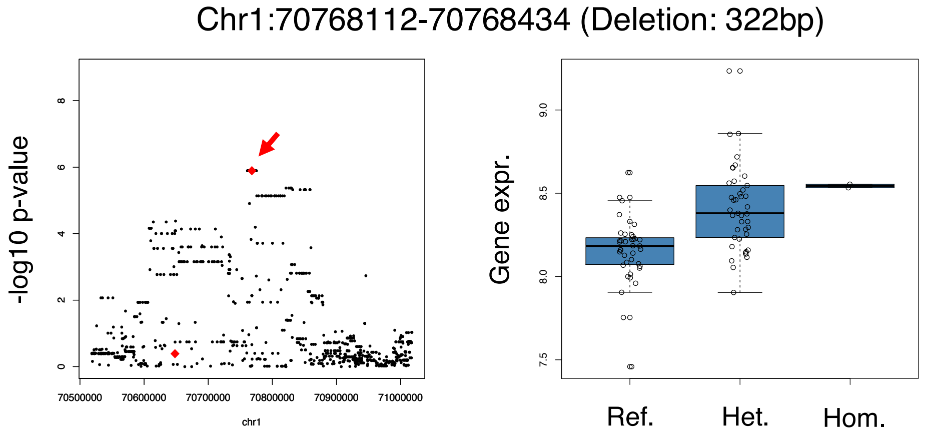
Chr1:70768112-70768434 (Deletion: 322bp)
-log10 p-value
0
2
4
6
8
70500000
70600000
70700000
70800000
70900000
71000000
chr1
Gene expr.
7.5
8.0
8.5
9.0
Ref.
Het.
Hom.

(b)

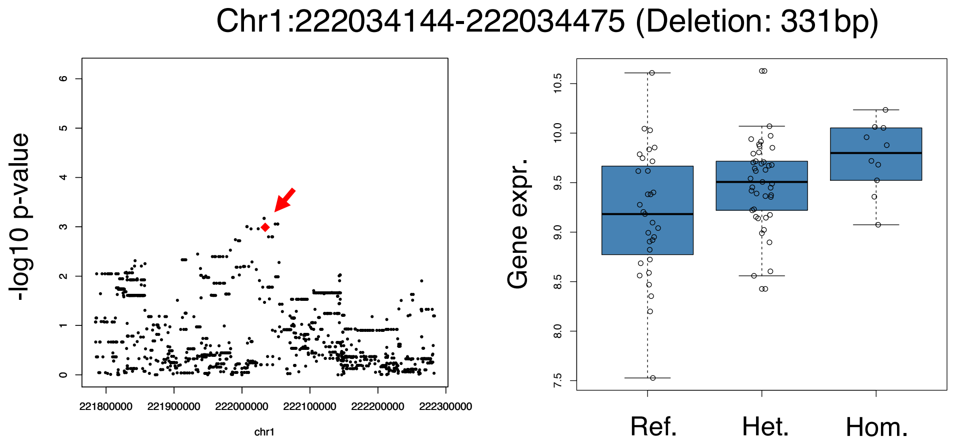
Chr1:222034144-222034475 (Deletion: 331bp)
-log10 p-value
0
1
2
3
4
5
6
221800000
221900000
222000000
222100000
222200000
222300000
chr1
Gene expr.
7.5
8.0
8.5
9.0
9.5
10.0
10.5
Ref.
Het.
Hom.

(c)

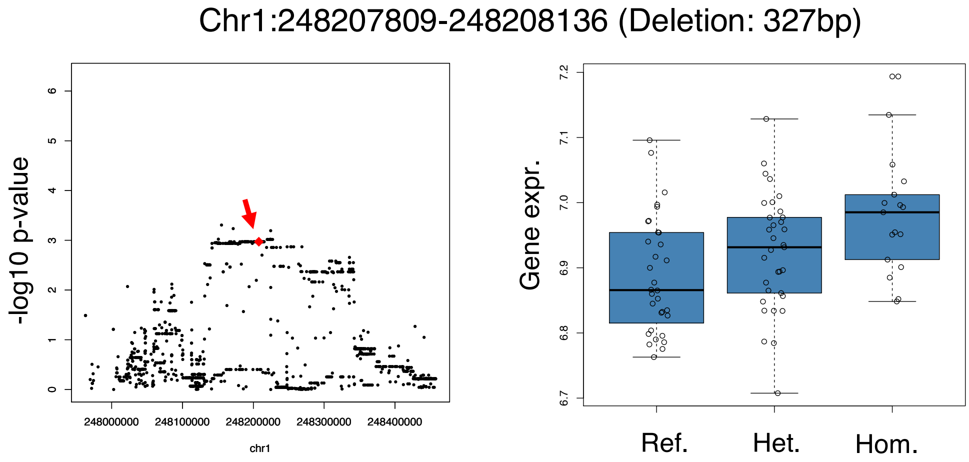
Chr1:248207809-248208136 (Deletion: 327bp)
-log10 p-value
0
1
2
3
4
5
6
248000000
248100000
248200000
248300000
248400000
chr1
Gene expr.
6.7
6.8
6.9
7.0
7.1
7.2
Ref.
Het.
Hom.

(d)

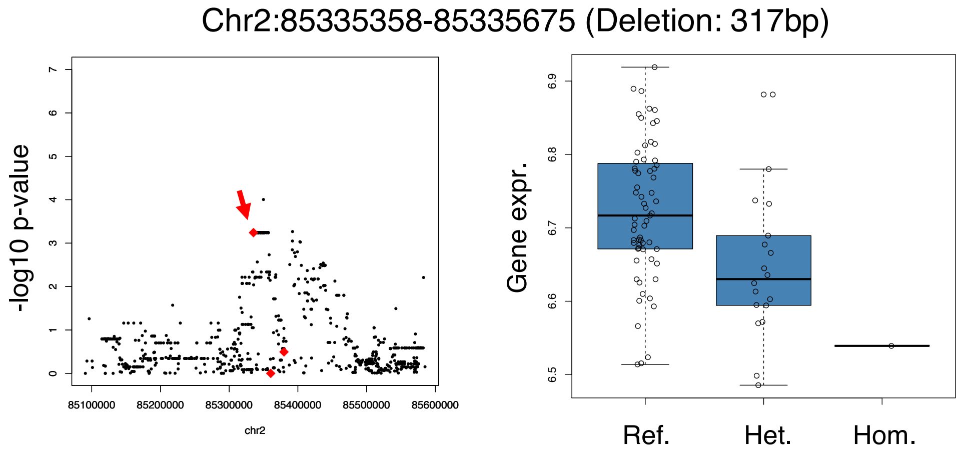
Chr2:85335358-85335675 (Deletion: 317bp)
-log10 p-value
85100000
85200000
85300000
85400000
85500000
85600000
chr2
Gene expr.
Ref.
Het.
Hom.

(e)

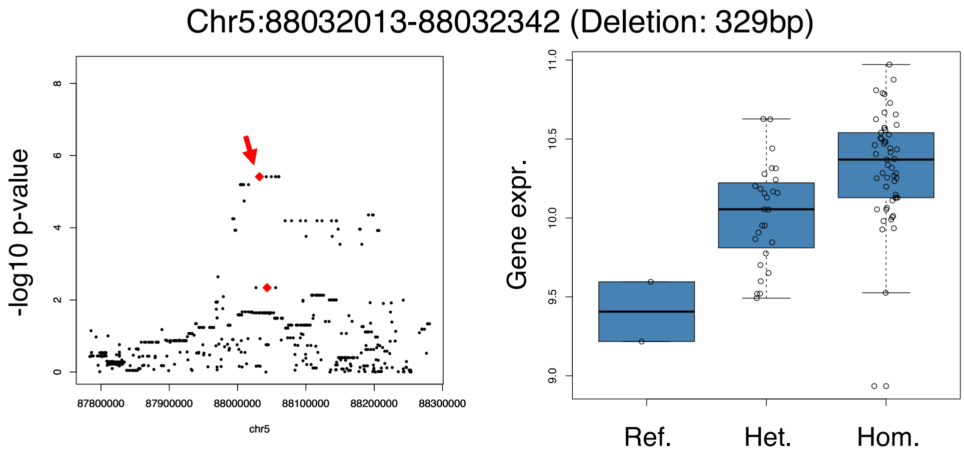
Chr5:88032013-88032342 (Deletion: 329bp)
-log10 p-value
87800000
87900000
88000000
88100000
88200000
88300000
chr5
Gene expr.
Ref.
Het.
Hom.

(f)

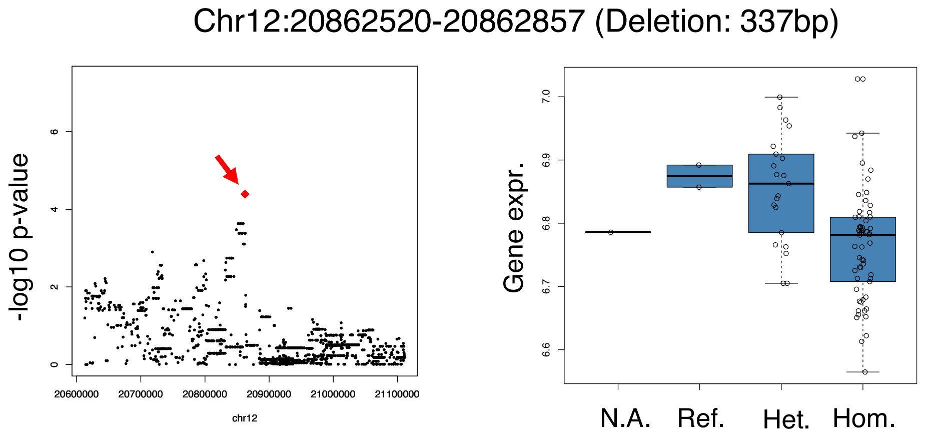
Chr12:20862520-20862857 (Deletion: 337bp)
-log10 p-value
20600000
20700000
20800000
20900000
21000000
21100000
chr12
Gene expr.
N.A.
Ref.
Het.
Hom.

(g)

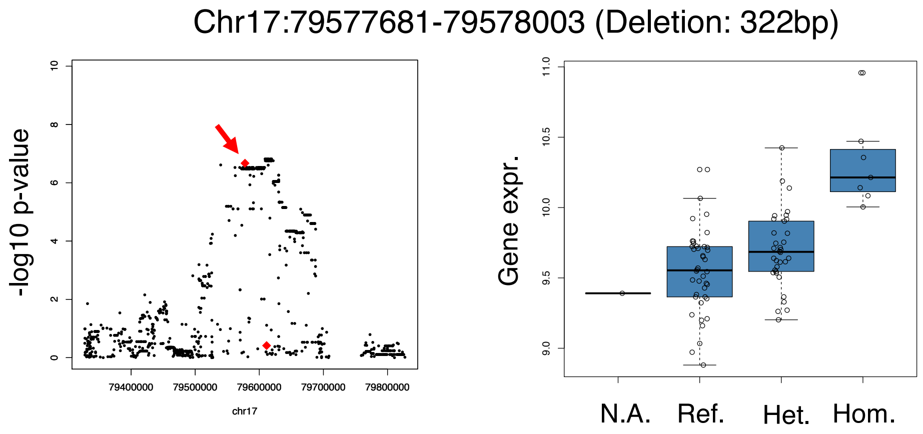

(h)

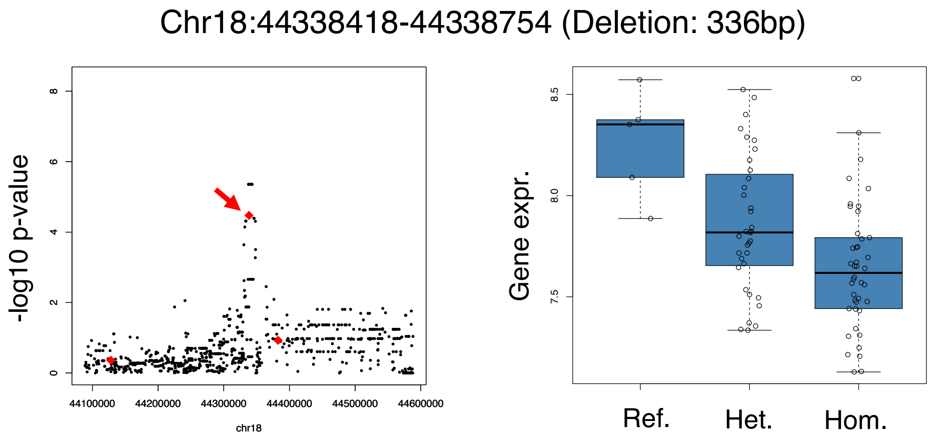

Figure S4: Examples of causal deletion candidates overlapping *Alu* transposons.

The figures show examples of the eQTL association p-value plots and gene expression boxplots of causal deletion candidates that overlapped *Alu* transposons. (a) Plots for 322bp deletion at chr1:70768112-70768434. (b) Plots for 331bp deletion at chr1:222034144-22034475. (c) Plots for 327bp deletion at chr1:248207809-248208136. (d) Plots for 317bp deletion at chr2:85335358-85335657. (e) Plots for 329bp deletion at chr5:88032013-88032342. (f) Plots for 337bp deletion at chr12:20862520-20862857. (g) Plots for 322bp deletion at chr17:79577681-79578003. (h) Plots for 336bp deletion at chr18:44338418-44338754. Deletion candidates in the region are shown as red diamonds in the eQTL association p-value plots, with red arrows indicating their positions. The x-axis shows their position on the respective chromosomes while the y-axis shows the –log10 of the eQTL association p-value.

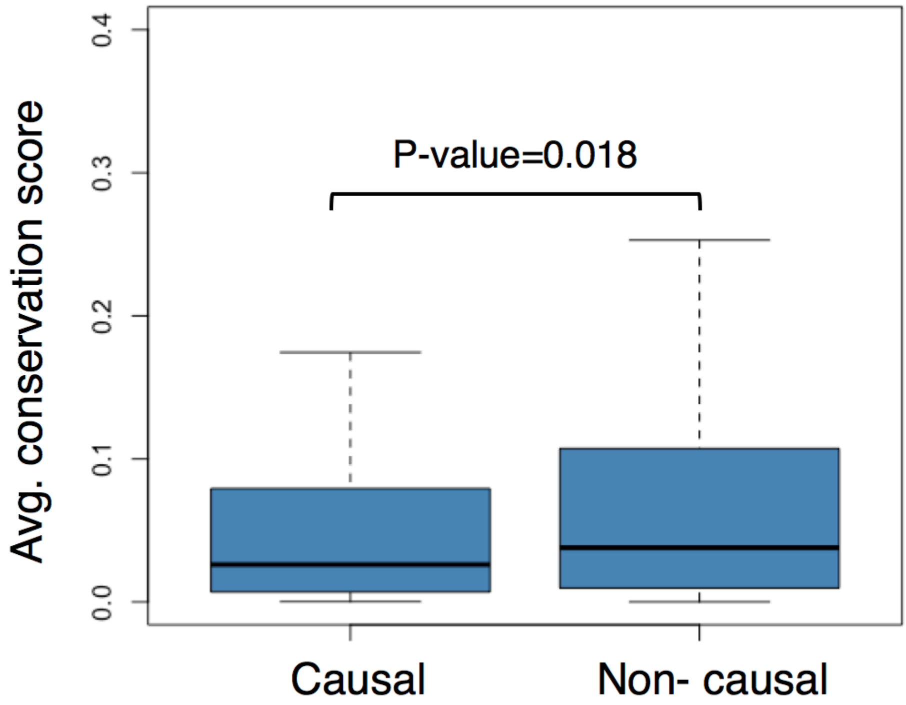

Figure S5: Average genome conservation score between causal and non-causal deletion candidates.

Enrichment of average genome conservation scores was seen between suggested causal deletion candidates and other deletion candidates, with causal deletion candidates seen to have lower average genome conservation scores compared to non-causal deletion candidates (Wilcoxon P-value=0.018).

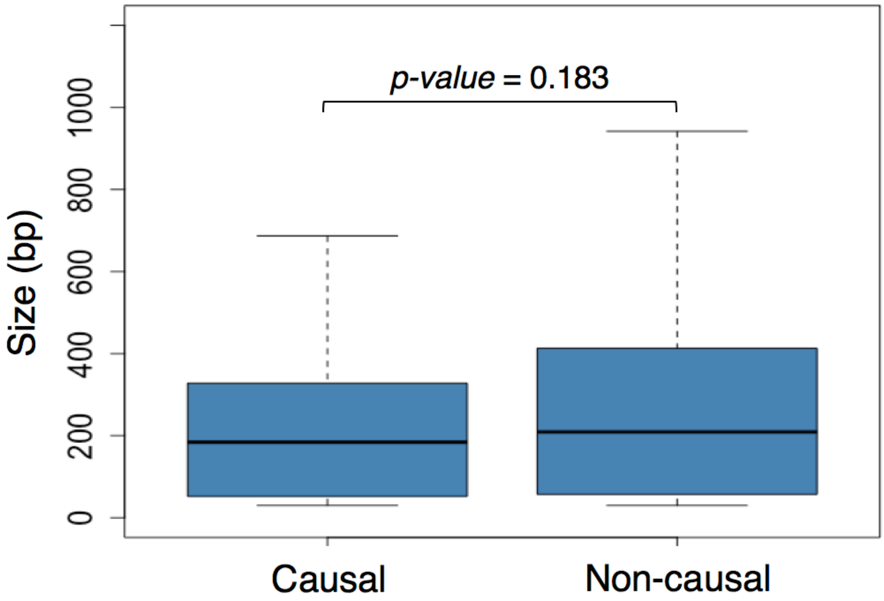

Figure S6: Size distribution between causal and non-causal deletions.

The distribution of deletion sizes between causal and non-causal deletion candidates. No significant difference (Wilcoxon Rank Sum test p-value=0.183) in deletion sizes was seen between casual and non-causal deletion candidates.

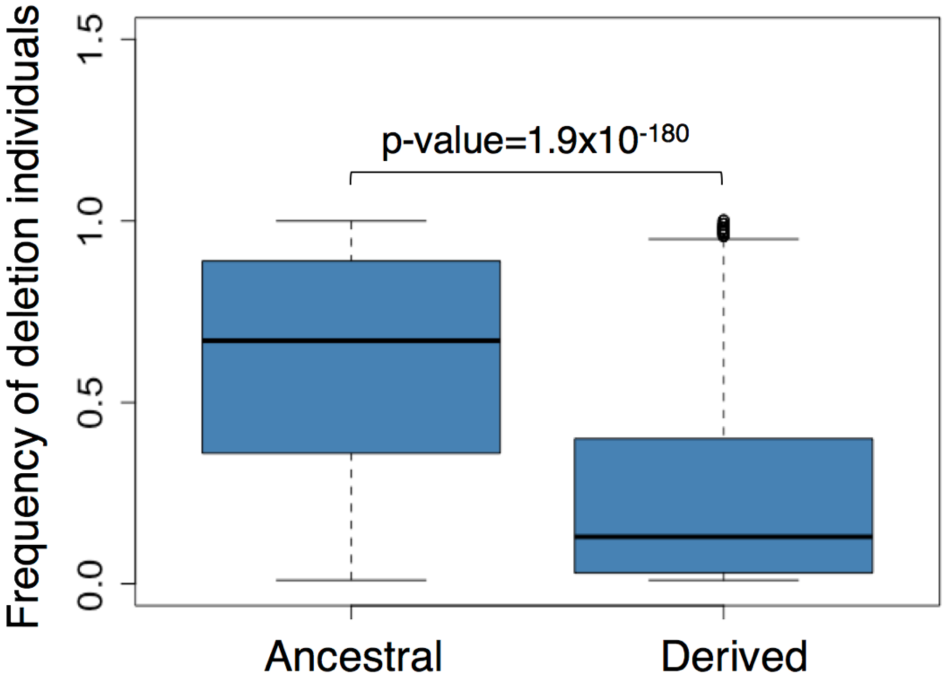

Figure S7: Frequencies of individuals with deletions for ancestral and derived deletion candidates.

Ancestral deletion candidates were seen to have significantly (Wilcoxon Rank Sum test p-value=$1.9x10^{-180}$) higher frequencies of individuals with deletions on average compared to derived deletion candidates.

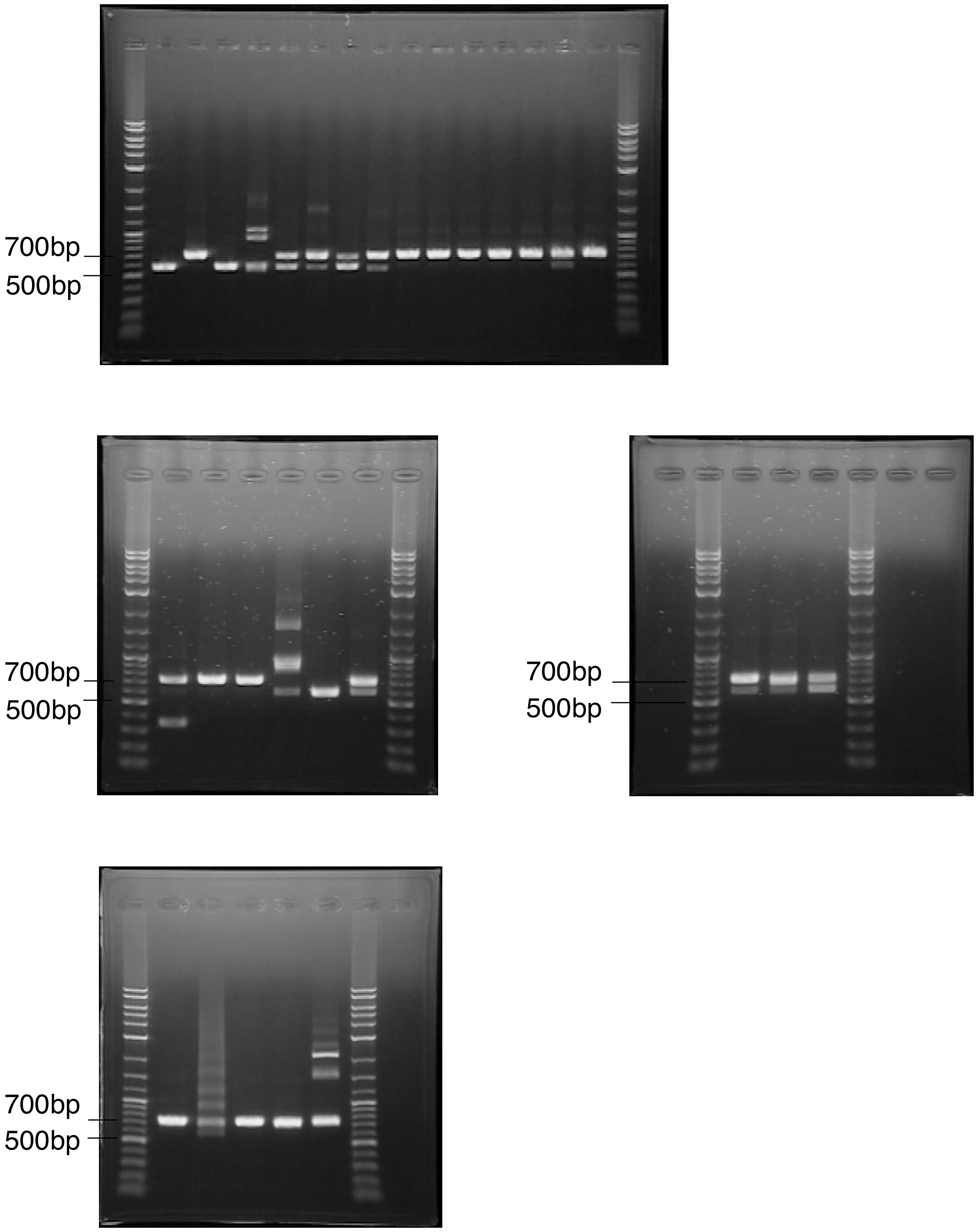

Figure S8: Gel electrophoresis of CRISPR-Cas9 induced deletions in HEK293T cells.
A deletion (chr9:130330770-130330813) was induced into HEK293T cells using the CRISPR-Cas9 system, and the presence or absence of deletion was confirmed in the cell clones using PCR and gel electrophoresis.

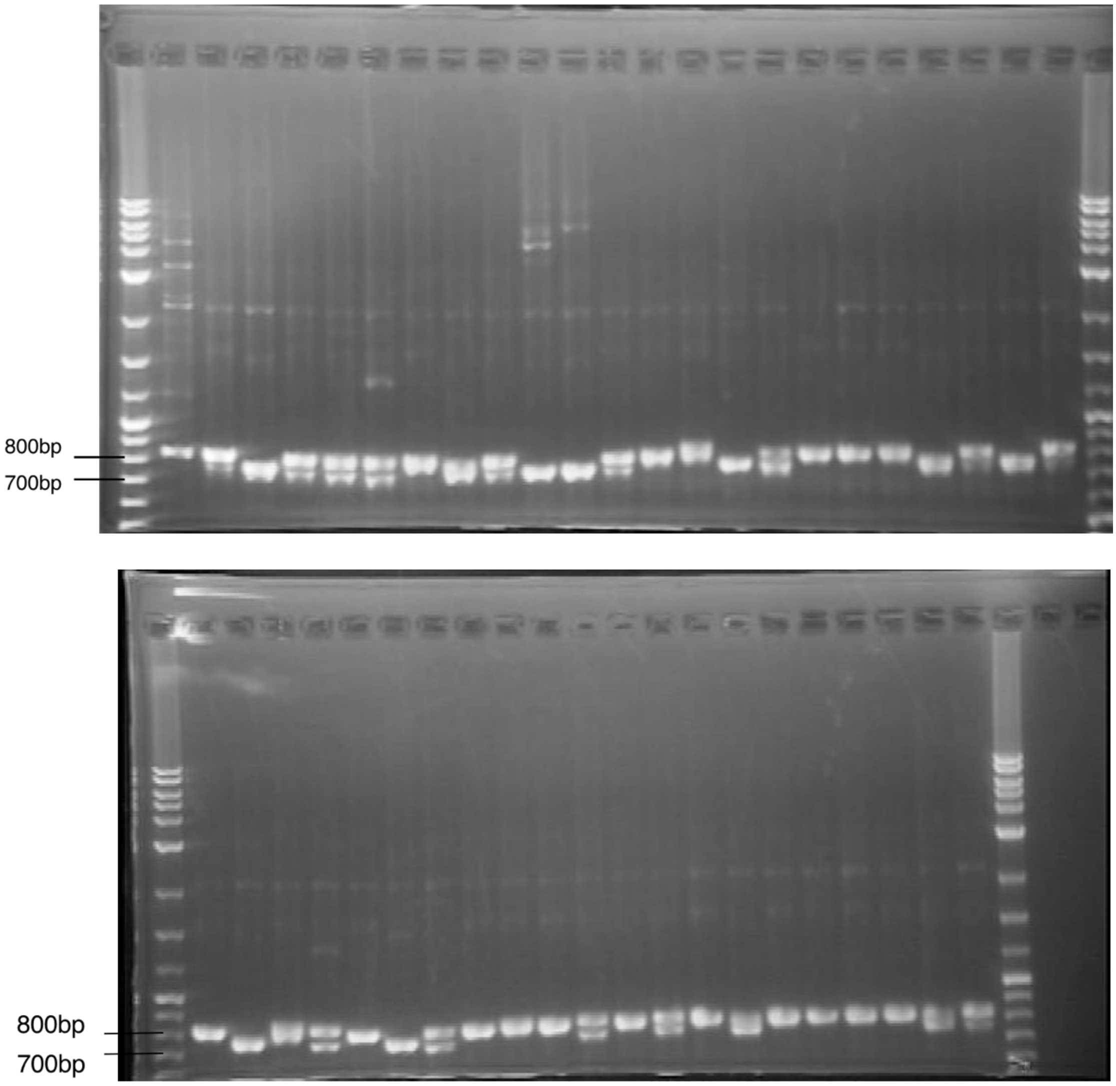

Figure S9: Gel electrophoresis of CRISPR-Cas9 induced deletions in HEK293T cells. A deletion (chr12:122230008-122230060) was induced into HEK293T cells using the CRISPR-Cas9 system, and the presence or absence of deletion was confirmed in the cell clones using PCR and gel electrophoresis.

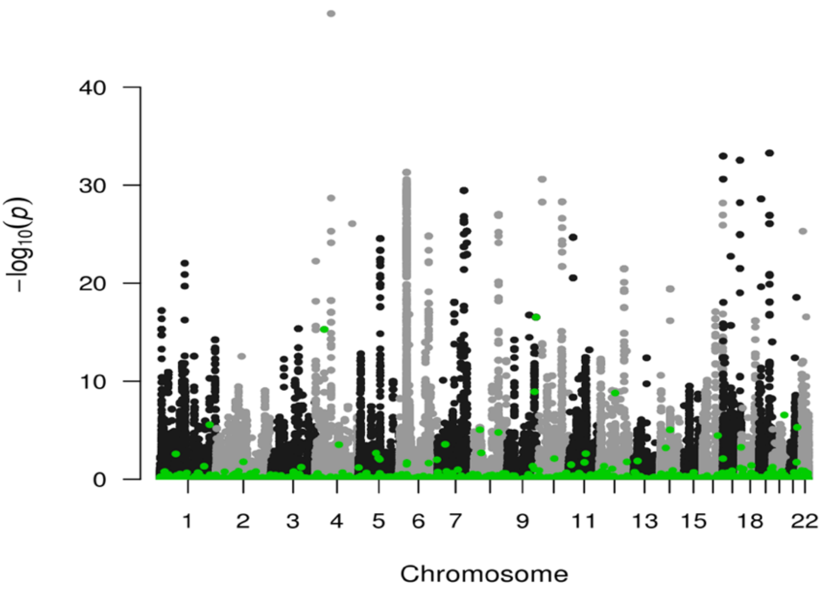

Figure S10. Manhattan plot of variants with identified intermediate-sized deletions highlighted in green. The x-axis shows the chromosome on which the variants are located in, while the y-axis is the –log10 of the lowest p-value for the variant in the eQTL association analysis.
