# Supplementary material for: Identification of intermediate-sized deletions and inference of their impact on gene expression in a human population
Source: Genome Med. 2019 Jul 24;11:44. doi: 10.1186/s13073-019-0656-4 (PMC6657090; doi:10.1186/s13073-019-0656-4)
Supplement: Supplementary file 2 — Supplementary note. File containing supplementary information. (PDF 142 kb) [file 13073_2019_656_MOESM2_ESM.pdf]

Supplementary note

Sample selection

Whole genome sequencing data from 202 Japanese samples from the ICCG Japanese samples were used in the current study [1]. Read sequences from these samples were mapped to the reference genome (GRCh37) and subsequently processed as described previously [1]. SNVs were called with VCMM software [2], and filtered by plink using the following conditions: (i) genotyping call rate $\geq$ 95%; (ii) minor allele frequency $\geq$ 5%; (iii) Hardy-Weinberg Equilibrium (HWE) p-value $\geq$ 0.000001.

A previous study suggests that the Japanese population can be classified into two major clusters; mainland and Ryukyu clusters [3]. To select mainland Japanese and exclude relatives for the subsequent analyses, we analyzed the SNVs. First, the proportion of homozygous and heterozygous SNVs was calculated for each sample, and samples with unusual proportions of homozygous and heterozygous SNVs were excluded from further analysis. Second, samples with cryptic relationships were identified using plink [4] (http://zzz.bwh.harvard.edu/plink/) and one sample from each pair identified as related was excluded. Third, a principal component analysis (PCA) was conducted to account for potential population stratification. The PCA was conducted using GCTA [5], with biallelic JPT and CHB SNP data from the 1000 Genomes project database [6] used as reference. Samples that were seen to lie outside the main Japanese cluster in the PCA were removed from further analysis. Finally, 174 samples were selected for the analysis.

Deletion detection by IMSindel, annotation and filtering of deletion candidates

As previous evaluations of IMSindel showed improved performance compared to other well-established SV detection software (GATK HaplotypeCaller, PINDEL, and ScanIndel) [7], we used IMSindel to detect deletions in the current study. Deletion candidates were detected

in each individual sample by IMSindel, and an overall deletion candidates set was created by combing all the candidates detected in the 174 Japanese WGS samples. Deletion candidates were merged into a single entry if their breakpoints were within 10bp of each other.

The overall deletion candidates set was annotated according to their positions in relation to genes, and positions of telomeric and centromeric regions, simple repeats, low-complexity regions, as well as microsatellite regions. Region lists used for the annotation were obtained from the UCSC Genome Brower (genome build: GRCh37/hg19) by using the Table Browser utility [8]. Gene locations used were from the "NCBI RefSeq" track in the "Genes and Gene Predictions" group in Table Browser, while centromere and telomere regions were obtained from the "gap" table under the hg19 database for "All Tables" group. Simple repeat regions were from the "Simple Repeats" track in the "Repeats" group, while low-complexity, microsatellite regions, as well as transposons (e.g. *Alu* and LINE transposons) were taken from the "RepeatMasker" track. The read depths of the deletion candidate regions and the flanking regions were obtained. Flanking regions were defined as the regions before and after the deletion candidates' breakpoints and were of equal length to the size of the deletion candidates. Soft-clipped bases' average quality scores were also calculated for the 110bp region before and after the breakpoints.

As the currently study sought to identify intermediate-sized deletions, we removed deletion candidates <30bp from further study. To filter the overall list of deletion candidates for potential falsely-identified deletions, we first annotated the candidates by comparing their intersections with the annotation regions, and deletions candidates were annotated according to their coverage of the regions. Deletion candidates would be classified as "within" an annotation region if their breakpoints were located between the start and end positions of the annotation region, "cover" if the start and end positions of the annotation region was located between the breakpoints (i.e. deletion candidate was larger than annotation region), or "partial"

if the overlap between candidates' breakpoints and annotation regions' start and end positions was not full. The proportion of overlap was also calculated for "partial" classifications. We also annotated the flanking regions (100bp) of deletion candidates in a similar manner.

To remove deletions which were likely to be mis-identified due to inaccurate mapping resulting from existing limitations of short-read sequencing technology in accurately mapping highly-repetitive regions, we firstly excluded candidates which were located within centromere or telomere regions. We next removed deletion candidates which were classified as "within" a simple repeat region, or within a microsatellite or low-complexity region, as well as deletion candidates classified as "partial" with proportions ≥50% for these regions. Furthermore, we decided to remove deletion candidates which had flanking regions that met the exclusion criteria as well, as the likelihood for these deletion candidates to have arose from inaccurate read mapping was high.

Additionally, we removed deletion candidates which had read depths of over 150 in the deletion region, as these were likely to be false positives resulting from a large number of reads inaccurately mapping to a repetitive region. As it is expected that read depths within deletion regions will be lower compared to the surrounding/flanking regions, we also excluded deletion candidates which were seen to have higher read depths within the deletion regions compared to the flanking regions. Furthermore, we expected that inaccurate mapping of reads would result in soft-clipped bases with low base quality scores, which would lead to IMSindel erroneously using these soft-clipped bases to call deletions. Thus, we calculated the average base quality score of soft-clipped bases in the 110bp region adjacent to deletion candidates' breakpoints, and removed deletion candidates which had average quality scores below 15.

To filter the deletion candidates by population filters (i.e Hardy-Weinberg Equilibrium (HWE) p-values), we obtained the counts of homozygous deletion alleles, heterozygous deletion alleles, and wild-type alleles for the samples and also calculated the expected counts

for each of these allele categories. HWE p-values for each deletion candidate was calculated using Fisher's exact test in R (version 3.3.3) and we excluded deletions which had HWE p-values <0.0001 from further analysis.

Application of joint-call recovery for deletion candidates and creation of accurate deletion candidates set

IMSindel makes use of information and quality of soft-clipped mapping reads to detect deletions [7], and consequently, the detected deletion candidates of each sample can be classified by the types of reads used for the detection of deletion. In particular, deletion candidates may be identified using only forward-oriented reads, only reverse-oriented reads, or using both read types. A fourth type of deletion candidate would be shorter and exists within a sequencing read, which we termed "within-type". We considered that deletion candidates that were identified using either forward- or reverse-type reads only were of lower quality and confidence compared to those identified using both reads types. To mitigate a loss of deletion detection sensitivity in samples with lower read depths or in which the deletion was called using only lower-confidence read types, we applied a joint-call recovery method to retain such deletions in these samples by leveraging the information of the same deletion in other samples.

Firstly, the deletion candidates were categorized according to their lengths into two main groups, the first being deletions that were in <50bp length and the second being those that were ≥50bp. For the first group, the candidate is retained if: (i) candidate is detected using forward-type reads in some samples and reverse-type reads in others; (ii) candidate is detected in some samples using within-type reads, while in other samples, it is detected using either forward-type, reverse-type, or both types of reads; or (iii) candidate is classified as within-type for all samples. For the second group, the candidate would be retained if: (i) candidate is detected using both forward- and reverse-type reads for all samples; (ii) candidate was detected

using forward-type reads in some samples, and reverse-type reads in other samples; (iii) candidate had ≥2 samples showing detection using both forward- and reverse-type reads, with at least 5 of such read types per sample, while also being detected in other samples using either forward-, reverse-, or both read types. Using these conditions, we were able to retain a deletion candidate, even if it was not identified using both forward- and reverse-type reads in some samples by leveraging information from other samples. This allowed for a reduction in the potential deletion candidates that would not be considered for further analysis and increased the detection sensitivity.

To further improve on the accuracy of the detected deletion candidates, we randomly selected 100 candidates and compared them to deletions detected using Oxford Nanopore long-reads sequencing. We observed a number of deletion candidates to be likely false positives and further inspection revealed that for these candidates, their breakpoints resided within low-complexity regions of the same class (e.g. same class of *Alu* transposons). We hypothesized that in such situations, the similarity of base sequences between the two regions may result in inaccurate mapping of sequencing reads, leading to inaccurate generation of soft-clipped bases, and false deletion detection by IMSindel. We thus excluded deletion candidates with such breakpoint patterns from our high-accuracy deletion candidates dataset, leading to 4,378 deletion candidates to be used for subsequent analysis and creation of the reference imputation panel.

WGS with Oxford Nanopore sequencer for assessing accuracy of deletion calls by IMSindel, effectiveness of processing steps, and accuracy of imputation

To evaluate our deletion calling and imputation accuracy, we sequenced whole genomes of two samples belonging to the mainland Japanese population group; RK067 from ICGC individuals and NA18943 from the 1000 Genomes project [1,9] using the Oxford

Nanopore long-reads sequencer. Library preparations were performed according to the manufacture's instruction. For sample RK067, 10 sequencing runs were performed, and 19 sequencing runs were performed for sample NA18943. Deletions supported by ≥ 2 uniquely mapped reads were used for the comparison with deletion calls from the short-reads sequencing data. These deletions were then used in subsequent comparisons with deletions detected by IMSindel.

To assess the accuracy of deletion detection by IMSindel as-well-as the effectiveness of the downstream filtering and processing steps, we compared the presence of deletion candidates detected by IMSindel with those detected using Nanopore sequencing at each of the processing steps. We compared the deletion candidates' breakpoint positions of IMSindel-detected candidates with those of Nanopore-detected deletions, for deletions ≥120bp in size. If the breakpoint positions of the deletions in both sets were within 100bp of each other (i.e. forward breakpoint of IMSindel-detected deletion candidate within 100bp of forward breakpoint of Nanopore-detected deletion, and the same was seen for reverse breakpoints), the deletion was considered to be in consensus between the two sets, and regarded as a true-positive deletion call. The true-positive rate was then taken as the consensus rate between IMSindel-detected deletion candidates and Nanopore-detected deletions.

Creation of imputation panel with intermediate-sized deletions

Data of SNVs from the previous Japanese WGS study was combined with the high-confidence deletion candidates to create a reference panel which was used for imputation. Firstly, we converted the SNVs information from the previous WGS in to Variant Call Format (VCF) and excluded variants if: (i) their minor allele frequency (MAF) <0.05; (ii) number of alleles >2; and HWE p-value <0.0001. Filtering was done using VCFtools (ver. 0.1.15) [10]. Information of the deletion candidates were also converted to VCF and merged with the WGS

variants' VCF to create an overall reference panel to be used for imputation (intermediate-sized deletions panel). In total, the reference panel contained 5,244,299 variants, including the 4,378 deletion candidates.

Imputation of intermediate-sized deletions panel into genotype data

Genotype data was obtained for 82 Japanese 1000 Genomes samples used in a previous study [11] from the 1000 Genomes Project database [6]. The 1000 Genomes samples' SNPs were filtered for those with MAF $\geq$0.05 and HWE p-values >0.00001. These SNPs were then compared against those in the intermediate-sized deletions panel, and SNPs that showed flipped strands between the two datasets were removed. Short indels were also removed from the 1000 Genomes dataset, together with SNPs from both datasets which were seen to have the same genomic positions but with different rsIDs or allele information.

The filtered intermediate-sized deletions panel was then imputed into the filtered genotype dataset of the 82 1000 Genomes Japanese samples using IMPUTE2 [12], with the following options (-k 100; -k_hap 700; -iter 40; -Ne 20000). Phasing of the datasets were done by IMPUTE2 with the "-phase" option and the 1000 Genomes dataset was split by chromosome and into 5Mb chunks during the imputation process. The genome map file used in the imputation was obtained from the IMPUTE2 website. The imputed deletion candidates and genotypes were then re-converted back to VCF using plink1.9 [4] (https://www.cog-genomics.org/plink2) with the hard call threshold for genotype possibilities set at 0.4999 to get as close as possible to 0.5. We estimated the accuracy of the imputation by comparing the consistency between the imputed deletion candidates of the Japanese 1000 Genomes sample NA18943 with corresponding detected deletions by Nanopore in the same sample. Presence of deletions between the two datasets were considered to be consistent if their breakpoints were located within 100bp of each other in each respective dataset.

Validation of imputed deletions

To evaluate the accuracy and efficacy of the imputation of deletion candidates into the Japanese samples, 11 imputed deletion candidates were randomly selected to be tested in 30 of the Japanese 1000 Genomes samples. Primers to capture the deletion candidate regions were designed using Primer3web (ver. 4.1.0) and polymerase chain reaction (PCR) was conducted to capture the deletion regions in the 30 samples. Briefly, the samples' DNA were obtained from the Coriell Institute and used for the PCR. The PCR was conducted using KOD -Multi & Epi- high-fidelity polymerase (Toyobo Life Sciences, Japan), with a pre-heating step at 98°C for 1 minute, followed by 35 cycles consisting of a denaturation step at 98°C for 15 seconds, a primer annealing step of 58°C for 30 seconds, and an elongation step of 68°C for 1 minute. A final elongation step at 68°C for 10 minutes was also used. The PCR was conducted using a SimpliAmp thermal cycler (Applied Biosystems/Thermo Fisher Scientific). The PCR products were visualized on 0.8% or 1.2% agarose S gels, depending on the product and deletion sizes. The results of the PCR were then checked against the estimated deletion type from the imputation results and the concordance rate was calculated.

eQTL association analysis and estimation of causal deletion candidates

Processed gene expression data for the 82 Japanese 1000 Genomes samples were downloaded from a publically-available database (EMBL-EBI ArrayExpress, see "URLs" section in main manuscript) and used together with the results of the imputation for eQTL association analysis. The transcription start site (TSS) positions of genes included in gene expression data were obtained using the Ensembl GRCh37 Release 93 biomart utility [13]. The eQTL mapping and association analysis was conducted using the MatrixEQTL R package. The imputation results and gene expression data were converted to the appropriate format for use

with MatrixEQTL. The cis-eQTL analysis was conducted using a linear model, with cis regions defined as 1Mb on both sides of gene TSS. The results of the eQTL association analysis underwent gene-level multiple-testing correction using the Benjamini-Hochberg (BH) method with false-discovery rate (FDR) 0.01 and deletions with $\geq$5% in the 1000 Genomes JPT samples were selected for further analyses.

The eQTL mapping and association analysis by MatrixEQTL found 217 deletion candidates that were significantly associated with gene expression level changes at q-value 0.01. From these significantly associated deletion candidates, we selected the most significantly associated deletion candidate and extracted the top 100 SNPs within a 250kb region around the deletion candidates, as well as any other deletion candidates that may be within the region. The genotypes of these variants were extracted from the original VCF files and r-values of linkage disequilibrium (LD) between these variants were calculated using PLINK1.9, while the p-values of the eQTL analysis for these variants were converted into Z-scores. We ran CAVIAR with causal set size of 1 for these regions using the z-scores and LD information (r values) of the extracted variants. For each tested region, the most significantly associated deletion candidate was estimated to be causal if it was present in the set of likely causal variants identified by CAVIAR. From the 217 significantly associated deletion candidates, 181 of them were seen to be suggested as causal by CAVIAR.

Annotation and enrichment analysis of regulatory features and average genome conservation scores

The deletion candidates were annotated according to their intersections with genomic regulatory features such as: (i) transcription factor binding sites; (ii) super enhancer sites; (iii) known regulatory features, such as promoters and enhancers; (iii) CTCF binding sites; (iv) microRNA (miRNA) binding sites; and (v) predicted chromatin states of the region. Briefly,

the data used for the annotation were obtained from the following sources: (i) for transcription factor ChIP data as well as predicted chromatin states from the ENCODE project/Broad, data was obtained from the UCSC Genome Browser database via the Table Browser [8]; (ii) super enhancer data was obtained from the super enhancer database dbSUPER [14] (http://asntech.org/dbsuper/); (iii) known regulatory features information and miRNA data from Ensembl GRCh37 Release 93 (https://grch37.ensembl.org/index.html); and (iv) CTCF binding sites data from the CTCFBSDB 2.0 database [15] (http://insulatordb.uthsc.edu/). Additionally, annotation was also done for regulatory regions and features specific to the GM12878 cell line. The GM12878-specific regulatory features used for annotation included: (i) transcription factor binding sites; (ii) super enhancer sites from dbSUPER; (iii) known regulatory features such as promoters, enhancers, CTCF binding sites, other transcription factor binding sites, and open chromatin regions from Ensembl GRCh37 Release 93; (iv) CTCF binding sites data from the CTCFBSDB2.0 database; as well as (v) ENCODE/Broad predicted chromatin states from the UCSC Genome Browser [16]. To calculate the average genome conservation scores for the high-confidence deletion candidates, we obtained genome conservation scores in phastCon format from the UCSC genome browser database [17] and for each deletion candidate, extracted the deletion region's per-base conservation score and calculated the average.

We next sought to determine whether there was enrichment of regulatory features for causal deletion candidates. Counts of each regulatory feature was obtained for suggested causal deletion candidates and compared to those of other deletion candidates by Fisher's exact test using R (version 3.3.3). We further divided the causal deletion candidates into two categories: (i) gene expression increasing deletion candidates; and (ii) gene expression decreasing deletion candidates, by means of the beta values output by MatrixEQTL. The two categories were compared against other using Fisher's exact tests by R (version 3.3.3). The average

conservation scores of causal deletion candidates with other deletion candidates was also compared using the Wilcoxon rank sum test to test for association between the average conservation score and causal deletion candidates.

We further endeavored to determine whether there was enrichment of overlapped *Alu* transposon regions among causal deletion candidates compared to non-causal deletion candidates. We considered a deletion candidate as an *Alu* transposon deletion if overlaps at least 90% of an *Alu* transposon annotation, resulting in a focus on deletion candidates of sizes 300bp-400bp. Deletion candidates that did not meet the criterion were defined as non-*Alu* deletions. The counts of causal and non-causal deletion candidates that overlapped *Alu* transposons were obtained, and Fisher's exact test was used for the enrichment analysis.

Enrichment analysis of phylogenetic status of deletion candidates

The list of the deletion breakpoints for the deletion candidates was obtained and the UCSC Liftover tool [18] was used to convert each breakpoint position from GRCh37/hg19 human genome build to the chimpanzee panTro5 build (http://hgdownload.cse.ucsc.edu/goldenpath/hg19/phastCons46way/). Deletion candidates were not included in the analysis if they met the following exclusion criteria: (i) liftover of deletion candidates' breakpoints was not successful; (ii) only one breakpoint position successfully converted; (iii) liftover chimpanzee chromosomes contained unordered chromosome sequences ("chrN_random") or clone contigs that were not in specific chromosomes ("chrUN"); and (iv) forward and reverse deletion candidates' breakpoints were placed in different chromosomes after liftover conversion. The region size of the corresponding chimpanzee genome was then calculated. The chimpanzee region was then compared to the deletion candidates' sizes. If the converted region in chimpanzee was larger than 80% of the deletion candidate's size, the deletion candidate was considered to be derived. Conversely,

deletion candidates were considered to be ancestral should the size of the converted chimpanzee genome region be less than 80% of the deletion candidate's size.

To conduct the enrichment analyses, the counts of ancestral and derived deletions for causal and non-causal deletion candidates were obtained and compared. We also obtained the counts of ancestral and derived deletions that overlapped *Alu* transposons and compared the frequencies to determine if there was enrichment of ancestral deletions among those that overlapped *Alu* transposons. The enrichment analyses were conducted by Fisher's exact test in R (version 3.3.3).

References

1. Fujimoto A, Furuta M, Totoki Y, Tsunoda T, Kato M, Shiraishi Y, et al. Whole-genome mutational landscape and characterization of noncoding and structural mutations in liver cancer. Nature Genetics. 2016;48:500–9.

2. Shigemizu D, Fujimoto A, Akiyama S, Abe T, Nakano K, Boroevich KA, et al. A practical method to detect SNVs and indels from whole genome and exome sequencing data. Scientific Reports. 2013;3:2161.

3. Yamaguchi-Kabata Y, Nakazono K, Takahashi A, Saito S, Hosono N, Kubo M, et al. Japanese Population Structure, Based on SNP Genotypes from 7003 Individuals Compared to Other Ethnic Groups: Effects on Population-Based Association Studies. The American Journal of Human Genetics. 2008;83:445–56.

4. Chang CC, Chow CC, Tellier LC, Vattikuti S, Purcell SM, Lee JJ. Second-generation PLINK: rising to the challenge of larger and richer datasets. GigaScience. 2015;4.

5. Yang J, Lee SH, Goddard ME, Visscher PM. GCTA: A Tool for Genome-wide Complex Trait Analysis. The American Journal of Human Genetics. 2011;88:76–82.

6. The 1000 Genomes Project Consortium, Gibbs RA, Boerwinkle E, Doddapaneni H, Han Y, Korchina V, et al. A global reference for human genetic variation. Nature. 2015;526:68–74.

7. Shigemizu D, Miya F, Akiyama S, Okuda S, Boroevich KA, Fujimoto A, et al. IMSindel: An accurate intermediate-size indel detection tool incorporating de novo assembly and gapped global-local alignment with split read analysis. Scientific Reports. 2018;8:5608.

8. Karolchik D. The UCSC Table Browser data retrieval tool. Nucleic Acids Research. 2004;32:493D – 496.

9. Fujimoto A, Nakagawa H, Hosono N, Nakano K, Abe T, Boroevich KA, et al. Whole-genome sequencing and comprehensive variant analysis of a Japanese individual using massively parallel sequencing. Nature Genetics. 2010;42:931–6.

10. Danecek P, Auton A, Abecasis G, Albers CA, Banks E, DePristo MA, et al. The variant call format and VCFtools. Bioinformatics. 2011;27:2156–8.

11. Stranger BE, Montgomery SB, Dimas AS, Parts L, Stegle O, Ingle CE, et al. Patterns of Cis Regulatory Variation in Diverse Human Populations. PLoS Genetics. 2012;8:e1002639.

12. Howie BN, Donnelly P, Marchini J. A Flexible and Accurate Genotype Imputation Method for the Next Generation of Genome-Wide Association Studies. PLoS Genetics. 2009;5:e1000529.

13. Kinsella RJ, Kahari A, Haider S, Zamora J, Proctor G, Spudich G, et al. Ensembl BioMarts: a hub for data retrieval across taxonomic space. Database. 2011;2011:bar030–bar030.

14. Khan A, Zhang X. dbSUPER: a database of super-enhancers in mouse and human genome. Nucleic Acids Research. 2016;44:D164–71.

15. Ziebarth JD, Bhattacharya A, Cui Y. CTCFBSDB 2.0: a database for CTCF-binding sites and genome organization. Nucleic Acids Research. 2012;41:D188–94.

16. Rosenbloom KR, Sloan CA, Malladi VS, Dreszer TR, Learned K, Kirkup VM, et al. ENCODE Data in the UCSC Genome Browser: year 5 update. Nucleic Acids Research. 2012;41:D56–63.

17. Casper J, Zweig AS, Villarreal C, Tyner C, Speir ML, Rosenbloom KR, et al. The UCSC Genome Browser database: 2018 update. Nucleic Acids Research. 2018;46:D762–9.

18. Hinrichs AS. The UCSC Genome Browser Database: update 2006. Nucleic Acids Research. 2006;34:D590–8.
